# Efflux Impacts Intracellular Accumulation Only in Actively Growing Bacterial Cells

**DOI:** 10.1128/mBio.02608-21

**Published:** 2021-10-12

**Authors:** Emily E. Whittle, Helen E. McNeil, Eleftheria Trampari, Mark Webber, Tim W. Overton, Jessica M. A. Blair

**Affiliations:** a College of Medical and Dental Sciences, Institute of Microbiology and Infection, University of Birmingham, Birmingham, United Kingdom; b Quadram Institute Bioscience, Norwich, United Kingdom; c School of Chemical Engineering, University of Birmingham, Birmingham, United Kingdom; McMaster University

**Keywords:** antibiotic resistance, efflux pumps, membrane permeability

## Abstract

For antibiotics with intracellular targets, effective treatment of bacterial infections requires the drug to accumulate to a high concentration inside cells. Bacteria produce a complex cell envelope and possess drug export efflux pumps to limit drug accumulation inside cells. Decreasing cell envelope permeability and increasing efflux pump activity can reduce intracellular accumulation of antibiotics and are commonly seen in antibiotic-resistant strains. Here, we show that the balance between influx and efflux differs depending on bacterial growth phase in Gram-negative bacteria. Accumulation of the fluorescent compound ethidium bromide (EtBr) was measured in Salmonella enterica serovar Typhimurium SL1344 (wild type) and efflux deficient (Δ*acrB*) strains during growth. In SL1344, EtBr accumulation remained low, regardless of growth phase, and did not correlate with *acrAB* transcription. EtBr accumulation in the Δ*acrB* strains was high in exponential phase but dropped sharply later in growth, with no significant difference from that in SL1344 in stationary phase. Low EtBr accumulation in stationary phase was not due to the upregulation of other efflux pumps but instead was due to decreased permeability of the envelope in stationary phase. Transcriptome sequencing (RNA-seq) identified changes in expression of several pathways that remodel the envelope in stationary phase, leading to lower permeability.

## INTRODUCTION

Antibiotic treatment failure in clinical infections is increasingly common due to the rise in multidrug resistant (MDR) Gram-negative bacteria. Infections with Gram-negative organisms are particularly difficult to treat due to their impermeable outer membranes and efflux pumps, which actively export antibiotic molecules out of the bacterial cell. Successful treatment relies on high concentrations of antibiotic accumulating within bacterial cells, which is a function of antibiotic influx and the rate of antibiotic efflux (1).

Small hydrophilic antibiotics such as β-lactams enter a Gram-negative bacterial cell through membrane pores called porins. The major porins of *Enterobacteriaceae* are OmpF and OmpC (2). Downregulation of porin genes contributes to antibiotic resistance by preventing antibiotics from entering the cell (3). In addition, mutations in the porin protein which change the channel diameter (4, 5) or the electric field inside the porin can block translocation of drugs across the membrane (5).

Some drugs can enter Gram-negative cells through the lipid outer and inner membranes via “self-promoted uptake.” This mechanism has been described for EDTA, polymyxin B, colistin and other cationic antimicrobial peptides (CAMPs), and aminoglycoside antibiotics (6–8). The chelator EDTA acts as a permeabilizer by displacing and chelating the cations (Mg^2+^ or Ca^2+^) that are essential for the stability of lipopolysaccharide (LPS) and the outer membrane (OM) (6, 9). CAMPs interact with anionic groups on lipid A, breaching the outer membrane, and cause poration in the inner membrane, leading to bacterial death.

*Enterobacteriaceae* contain efflux pumps from 6 classes. Major facilitator superfamily (MFS), small multidrug resistance family (SMR), multidug and toxic compound extrusion (MATE), resistance-nodulation-division (RND), and the recently described proteobacterial antimicrobial compound efflux family (PACE) pumps (10) utilize the proton motive force for export of molecules such as antibiotics, and ABC (ATP binding cassette) pumps utilize ATP hydrolysis. RND pumps are commonly upregulated in clinical isolates and can contribute to resistance to a number of antibiotic classes, as well as dyes, detergents, and biocides (11). The best-described RND pump is AcrAB-TolC, found in Salmonella enterica serovar Typhimurium and Escherichia coli. As efflux pumps underpin antibiotic resistance in essentially all bacteria of clinical and veterinary importance (12, 13), there is ongoing active research into the development of efflux inhibitors to potentiate the action of existing antibiotics.

Previous studies undertaken with cells in exponential growth phase have highlighted the importance of efflux pumps in minimizing intracellular drug accumulation (14–17). However, transcription of *acrAB* is growth phase dependent, with a peak in mid-exponential phase, which drops as cells enter stationary phase (18). The importance of AcrAB-TolC in bacterial cells in stationary phase which are slow growing or nongrowing is not known. However, it has been suggested that whereas survival of exponential-phase E. coli following treatment with the anionic detergent sodium dodecyl sulfate (SDS) is dependent on efflux, stationary-phase cell survival is efflux independent and is instead mediated by decreased permeability of the bacterial cell envelope, directed by the stationary-phase sigma factor RpoS (19). Little is known about the balance between influx and efflux in different growth phases and how this may relate to different growth states that may occur in an infection.

Previous studies have shown that the E. coli envelope changes in stationary phase compared to logarithmic growth, and it is possible that this could alter antibiotic influx in nongrowing bacterial cells. Outer membrane changes include a decrease in the overall concentration of membrane proteins (20) and an increase in lipoprotein cross-linked to peptidoglycan (21, 22) to strengthen the outer barrier. In the inner membrane, the composition of fatty acids changes with a decrease in monounsaturated fatty acids (23) and an increase in cyclopropane fatty acids, catalyzed by Cfa (24). Increased layers of peptidoglycan have also been observed in stationary phase (25).

Using a combination of fluorescent drug accumulation assays (17) and measurement of efflux gene transcription in wild-type and efflux mutant strains, we assessed the importance of the balance between influx and efflux in different growth phases in Gram-negative bacteria, using the model organism Salmonella enterica serovar Typhimurium. We also used RNA sequencing (RNA-seq) to measure the global transcriptome as bacteria enter stationary phase and correlate transcriptomic changes with biochemical and physiological changes in the cell envelope that lead to alterations in permeability.

## RESULTS

### Intracellular EtBr accumulation by *S*. Typhimurium is independent of growth phase-dependent *acrAB* transcription.

Using a recently developed flow cytometry method (17), both the intracellular accumulation of the fluorescent dye ethidium bromide (EtBr) and the transcription of *acrAB* (via a promoter-green fluorescent protein [GFP] fusion) were measured in parallel in single cells of Salmonella grown in drug-free medium. Samples were taken hourly during batch culture before EtBr was added to the sample immediately prior to flow cytometry analysis to measure accumulation (EtBr was not present in growing the culture).

**FIG 1:**
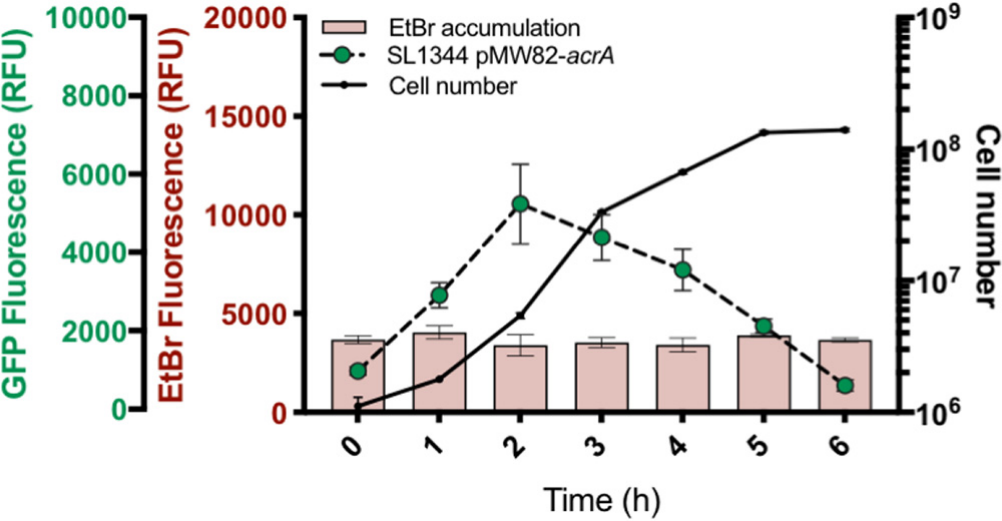
Ethidium bromide accumulation and *acrAB* expression in single cells of *S*. Typhimurium SL1344 across the growth phase. Cell number per milliliter was measured in each sample (black lines; numbers indicated on the right *y* axis). Pink bars indicate median ethidium bromide fluorescence per cell (relating to left red *y* axis), and dashed lines with green circles show *acrAB* expression (median GFP fluorescence per cell from the reporter; left green *y* axis). All data points are median values from measurements of 10,000 single cells of SL1344. Error bars indicate standard errors of the means (SEM).

Transcription of *acrAB* in SL1344 was growth phase dependent and peaked in early to mid-log phase before decreasing toward stationary phase (Fig. 1), as previously described (18). Previous studies have shown that increased expression of *acrAB* in clinical isolates leads to decreased susceptibility to antibiotics (12). Given the known role of efflux pumps in drug export, one might predict that EtBr accumulation would be lowest when efflux expression was highest. Our data, however, show that this is not the case. In SL1344 cells, accumulation of EtBr was low and remained unchanged across growth despite changes in *acrAB* transcription (Fig. 1). Therefore, changes in efflux pump transcription in different growth phases do not alter levels of accumulation within the cell.

### Growth phase-dependent transcription of *acrAB* does not correlate with intracellular EtBr accumulation, efflux capacity, or AcrAB protein level.

Having shown that *acrAB* transcription does not correlate with ethidium bromide accumulation, the efflux function in a population of cells was measured to determine whether efflux activity varied with growth phase (and *acrAB* expression), even if accumulation did not.

**FIG 2:**
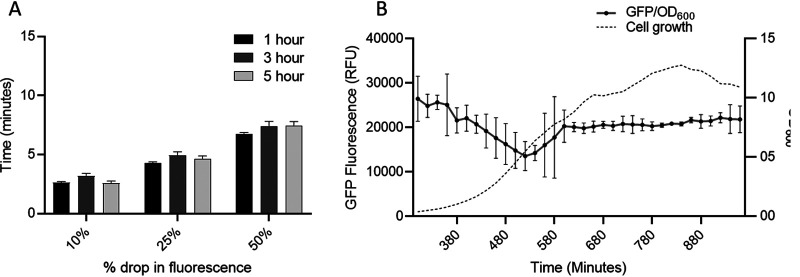
(A) Time taken for ethidium bromide to be removed from SL1344 cells at 1, 3, and 5 h. Bars represent the time taken for ethidium bromide fluorescence to drop 10%, 25%, and 50% from its original value. Data are based on 3 biological replicates, with error bars showing SEM. Data at 1 h (black), 3 h (dark gray), and 5 h (light gray) are shown. There was no significant difference in the time taken to export EtBr at each time point. (B) GFP/OD_600_ from SL1344 AcrB-GFP over 16 h of growth in MOPS minimal medium. This graph shows GFP/OD_600_ from AcrB-GFP at the end of lag phase (300 min) until the last time point at 16 h. The dashed black line shows the OD_600_; the green line shows GFP fluorescence (error bars show SEM). SL1344 autofluorescence was subtracted from these data.

To measure functional efflux capacity of cells, we used the previously described direct efflux activity assay (14), which was further optimized to analyze efflux capacity at three different time points across growth in SL1344. This assay determines the efflux capacity of the cell based on the activity of all efflux pumps (not just AcrAB-TolC) that are able to transport EtBr. Cultures grown for 1, 3, and 5 h had the same capacity for efflux of the substrate, as there was no significant difference in efflux rate between samples taken at each time point (Fig. 2A) (based on time taken for ethidium bromide fluorescence to drop 10%, 25%, and 50% from its maximum fluorescence value) regardless of the different levels of *acrAB* transcription at these time points already established.

Taken together, the low accumulation and similar rate of efflux of EtBr across time in SL1344 suggests that although *acrAB* transcription peaks in mid-exponential phase, activity of the assembled AcrAB-TolC complex remains constant. The AcrB protein is known to be very stable once made, with a predicted half-life of 6 days (26). To measure AcrB protein level at different points during growth, a strain was constructed in which the AcrB protein was tagged with GFP at the C terminus as previously described (27). The generation time and efflux level in this strain were unaffected, confirming that tagging the C terminus of AcrB with GFP did not affect its function. Measurement of GFP fluorescence during 16 h of growth showed that AcrB level remains constant (Fig. 2B). These data suggest that efflux capacity is constant regardless of growth phase due to the constant level of AcrAB protein within a population and may explain why EtBr accumulation remained low in stationary phase despite decreased efflux gene transcription.

### Intracellular accumulation is dependent on efflux only in actively growing cells.

**FIG 3:**
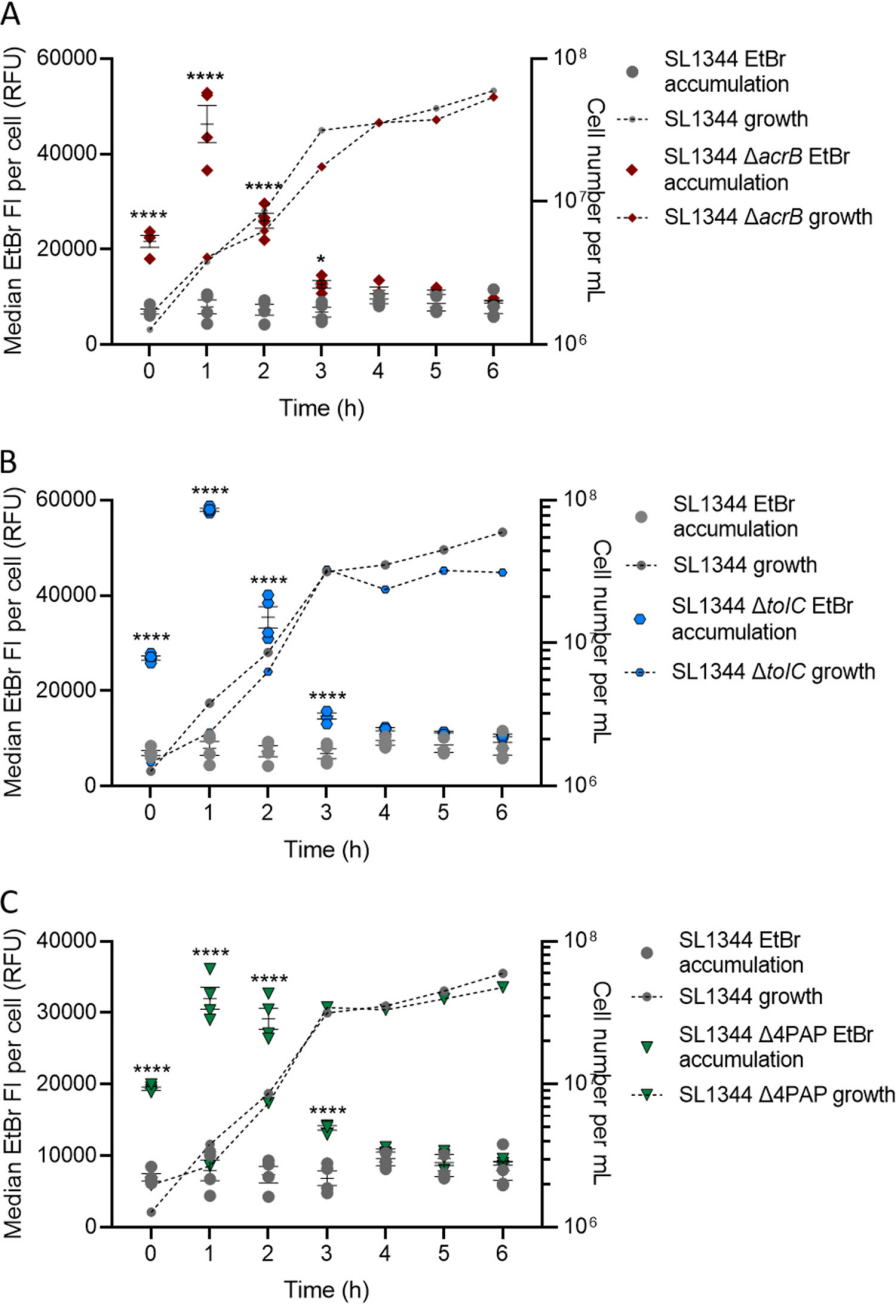
EtBr accumulation in SL1344 and SL1344 Δ*acrB* (A), Δ*tolC* (B), and Δ4PAP (C). For each strain, the median EtBr fluorescence per cell in 10,000 single cells was measured every hour between 0 and 6 h of growth for SL1344 (gray circles) and (A) SL1344 Δ*acrB* (red diamonds), (B) SL1344 Δ*tolC* (blue hexagons), and (C) SL1344 Δ4PAP (Δ*acrA* Δ*acrE* Δ*mdsA* Δ*mdtA*) (green triangles). Data from 4 biological replicates for each strain are shown; horizontal bars show means, and error bars show SEM. Median EtBr fluorescence per cell is plotted on the left *y* axis. Calculated cell numbers per milliliter are plotted on the right *y* axis with corresponding symbols equating to strain and a dashed line to show growth of the cultures. Cell numbers were based on the mean of the same biological replicates and the same gated population that EtBr fluorescence was measured from. Two-way analysis of variance (ANOVA) and Sidak’s multiple-comparison test were carried out for statistical analysis. At 0, 1, and 2 h, EtBr accumulation was significantly increased in Δ*acrB* with *P* values of <0.0001 (****). At 0, 1, 2, and 3 h, EtBr accumulation was significantly increased in SL1344 Δ*tolC* and SL1344 Δ4PAP, with *P* values of <0.0001.

To further dissect the importance of efflux during different growth stages, we measured EtBr accumulation (as for Fig. 1) in the presence or absence of AcrAB-TolC function (using SL1344 Δ*acrB*). The data in Fig. 2 suggest AcrAB-TolC activity is constant; therefore, it was assumed that by removing the efflux pump, EtBr accumulation would be high across growth. When EtBr accumulation in SL1344 Δ*acrB* was measured after 1 h of growth, it was 6-fold higher than in SL1344. This is similar to the growth time point used in most other published studies that have shown an increase in accumulation upon deletion of *acrB* (11, 14, 15, 17). However, EtBr accumulation then decreased dramatically and was not significantly different from that of the wild type (WT) from 3 to 6 h of growth (Fig. 3A). This suggests that low accumulation at 1 h in SL1344 greatly depends on efflux to export ethidium bromide from actively growing cells. As there is no significant difference between Δ*acrB* and WT cells from 3 to 6 h, it suggests that AcrAB-TolC is not important in maintaining low accumulation in slower-growing or stationary-phase cells. This is also supported by the *acrAB* expression data, which show highest expression in the early stages of logarithmic growth.

To confirm that low EtBr accumulation in stationary phase was not due to the activity of other RND efflux pumps present in SL1344, EtBr accumulation was also measured in two other mutants of SL1344. In the first, *tolC* was deleted, which compromised most efflux systems in Salmonella which use TolC as a common outer membrane channel. The second strain used lacked all four periplasmic adaptor proteins (Δ4PAP; Δ*acrA* Δ*acrE* Δ*mdsA* Δ*mdtA*) and is incapable of assembling any functional RND efflux systems. In both strains, the EtBr accumulation pattern observed recapitulated that seen in SL1344 Δ*acrB*, with a peak in accumulation at 1 h but no significant difference from that of SL1344 in stationary-phase cells (Fig. 3B and C). This result showed that low accumulation in stationary phase was not due to any RND pump in SL1344 (or the ABC pump MacAB-TolC). In addition, we also showed that, apart from *acrAB*, whose transcription was highest in mid-log phase and lowest in stationary phase, no other RND pump was actively transcribed under the conditions used to measure accumulation capacity across growth (see Fig. S1 in the supplemental material). For pumps from other families, only *macA* (ABC), *mdfA* (MFS) and *mdtK* (MATE) were transcribed and only at low levels (Fig. S1).

10.1128/mBio.02608-21.1 FIG S1 Transcription of efflux pumps across growth using GFP transcription reporters. In graphs A to I, the GFP/OD_600_ (measured using a plate reader) and therefore transcription of each pump is represented as a solid blue line (SL1344) or a dashed blue line (SL1344 Δ*acrB*). The OD_600_ is shown as a solid black line (SL1344) or a dashed black line (SL1344 Δ*acrB*). Each graph represents the fluorescence of a different transcriptional reporter: (A) *acrA*, (B) *acrE*, (C) *acrD*, (E) *mdsA*, (D) *mdtA*, (F) *emrA*, (G) *mdfA*, (H) *mdtK*, and (I) *macA*. Download FIG S1, TIF file, 0.2 MB. Copyright © 2021 Whittle et al. 2021 Whittle et al. https://creativecommons.org/licenses/by/4.0/ This content is distributed under the terms of the Creative Commons Attribution 4.0 International license.

Further investigation into the role of efflux pumps in stationary-phase EtBr accumulation was carried out by measurement in the presence of the proton motive force inhibitor CCCP (carbonyl cyanide *m*-chlorophenylhydrazone). Inhibiting the proton motive force inhibits the activity of the RND, MFS, and MATE pumps of SL1344 (28, 29). In SL1344 in the presence of CCCP, EtBr accumulation peaked at 1 h (Fig. S2). Accumulation levels started to drop in stationary phase, strikingly similar to those in SL1344 Δ*acrB*, again suggesting that low accumulation in stationary phase is not dependent on RND-, MFS-, or MATE-mediated efflux. This independent confirmation using different mutants and inhibitors demonstrates that the observed low EtBr accumulation in stationary phase is efflux independent.

10.1128/mBio.02608-21.2 FIG S2 EtBr accumulation in SL1344 plus 100 μM CCCP. Median EtBr fluorescence per cell in 10,000 SYTO-84^+^ flow cytometry events was measured every hour between 0 and 6 h. Individual green circles (WT) and individual green diamonds (+CCCP) represent the median value of EtBr fluorescence within a biological replicate. Two biological replicates for the control strains are shown, and 4 replicates for those with CCCP, with a short mean bar and error bars showing SEM. EtBr accumulation is plotted on the left *y* axis. Calculated cell numbers are plotted on the right *y* axis with corresponding symbols equating to strain and a dashed line to show growth of the culture. Cell numbers were based on the mean of the same biological replicates and the same gated population that EtBr fluorescence was measured from. Two-way ANOVA and Sidak’s multiple comparisons test were used for statistical analysis; At 0, 1, 2, 3, and 5 h, EtBr accumulation was significantly increased in the presence of CCCP with *P* values of <0.0001 (****). At 4 h and 6 h, EtBr accumulation was significantly increased with *P* values of 0.0003 (***) and 0.0002 (***), respectively. Download FIG S2, TIF file, 0.08 MB. Copyright © 2021 Whittle et al. 2021 Whittle et al. https://creativecommons.org/licenses/by/4.0/ This content is distributed under the terms of the Creative Commons Attribution 4.0 International license.

To investigate whether this was just a Salmonella phenomenon, EtBr accumulation was measured in wild-type and a mutant lacking the major RND efflux pump of other Gram-negative bacterial species, including Escherichia coli (MG1655 and MG1655 Δ*acrB*), Pseudomonas aeruginosa (PAO1 and PAO1 Δ*mexA*), and Klebsiella pneumoniae (ecl8 and ecl8 *acrB*::Gm). In E. coli and K. pneumoniae, EtBr accumulation was low throughout growth for the wild type but peaked at 1 h for each *acrB* mutant (Fig. S3), and in P. aeruginosa, the *mexA* mutant peaked at 2 h (Fig. S4) and then dropped to WT levels in stationary phase. Therefore, very similar observations are seen in a wide range of Gram-negative organisms.

10.1128/mBio.02608-21.3 FIG S3 EtBr accumulation in E. coli and K. pneumoniae efflux mutants. (A) K. pneumoniae ecl8 and ecl8 *acrB*::Gm. Median EtBr fluorescence per cell in 10,000 SYTO-84^+^ flow cytometry events was measured every hour between 0 and 6 h. Individual blue circles (WT) and individual purple diamonds (*acrB*::Gm) represent the median values of EtBr fluorescence within a biological replicate. (B) E. coli MG1655 and MG1655 Δ*acrB*. Median EtBr fluorescence per cell in 10,000 SYTO-84^+^ flow cytometry events was measured every hour between 0 and 6 h. Individual black Xs (WT) and individual blue triangles (Δ*acrB* strain) represent the median values of EtBr fluorescence within a biological replicate. Four biological replicates for each strain are shown, with a short mean bar and error bars showing SEM. EtBr accumulation is plotted on the left *y* axis. Calculated cell numbers are plotted on the right *y* axis with corresponding symbols equating to strain and a dashed line to show growth of the culture. Cell numbers were based on the mean of the same biological replicates and the same gated population that EtBr fluorescence was measured from. Two-way ANOVA and Sidak’s multiple-comparison test were carried out for statistical analysis. In K. pneumoniae, EtBr accumulation is significantly increased in *acrB*::Gm at 0, 1, 2, 3, 4, and 5 h with *P* values of <0.0001 (****). In E. coli, EtBr accumulation is significantly increased in the Δ*acrB* mutant at 1 and 2 h with *P* values of <0.0001 (****). Download FIG S3, TIF file, 0.2 MB. Copyright © 2021 Whittle et al. 2021 Whittle et al. https://creativecommons.org/licenses/by/4.0/ This content is distributed under the terms of the Creative Commons Attribution 4.0 International license.

10.1128/mBio.02608-21.4 FIG S4 EtBr accumulation in P. aeruginosa. Median EtBr fluorescence per cell in 10,000 SYTO-84^+^ flow cytometry events was measured every hour between 0 and 6 h. Individual green circles (WT) and individual blue hexagons (Δ*mexA*) represent the median value of EtBr fluorescence within a biological replicate.4 biological replicates for each strain are shown, with a short mean bar and error bars showing SEM. EtBr accumulation is plotted on the left *y* axis. Calculated cell numbers are plotted on the right *y* axis with corresponding symbols equating to strain and a dashed line to show growth of the culture. Cell numbers were based on the mean of the same biological replicates and the same gated population that EtBr fluorescence was measured from. EtBr accumulation is significantly increased in the Δ*mexA* strain with *P* values of <0.0001 (****) at 1, 2, and 3 h and 0.0024 (**) at 5 h. Download FIG S4, TIF file, 0.07 MB. Copyright © 2021 Whittle et al. 2021 Whittle et al. https://creativecommons.org/licenses/by/4.0/ This content is distributed under the terms of the Creative Commons Attribution 4.0 International license.

The EtBr accumulation pattern in Salmonella was also shown in MOPS (morpholinepropanesulfonic acid) minimal medium, suggesting that the pattern was not influenced by medium type and specifically was not a result of the limitations of LB (30) (Fig. S5). Even though it is a well-established and -studied model efflux substrate, to counter the possibility that EtBr would give abnormal results which are not representative of other efflux substrates, the same accumulation pattern in Salmonella WT and Δ*tolC* strains was also shown using the lipophilic dye Nile red (Fig. S6). Unlike EtBr, Nile red fluoresces not on intercalation with DNA but rather when bound to phospholipids or triglycerides (31), showing that this is not an artifact of the dye initially used. Together, these data show that the accumulation pattern described in the absence of efflux is consistent regardless of Gram-negative species, medium type, or efflux substrate used.

10.1128/mBio.02608-21.5 FIG S5 EtBr accumulation in SL1344 and SL1344 Δ*acrB* grown in MOPs minimal media. Median EtBr fluorescence per cell in 10,000 SYTO-84^+^ flow cytometry events was measured every hour between 0 and 6 h. Individual black circles represent the X-median value of EtBr fluorescence in SL1344 (WT) (from 10,000 SYTO-84^+^ cell events) within a biological replicate and, therefore, the average accumulation of ethidium per cell. Individual pink diamonds represent the X-median values of EtBr fluorescence in SL1344 Δ*acrB* (from 10,000 SYTO-84^+^ cell events) within a biological replicate. Four biological replicates for each strain are shown, with a short mean bar and error bars showing SEM. EtBr accumulation is plotted on the left *y* axis. Calculated cell number values are plotted on the right *y* axis with corresponding symbols equating to strain and a dashed line to show growth of the culture. Cell numbers were based on the mean of the same biological replicates and the same gated population that EtBr fluorescence was measured from. Two-way ANOVA and Sidak’s multiple-comparison test were carried out for statistical analysis. *P* values were <0.0001 (****) at 0, 1, 2, 3, and 4 h, 0.0003 (***) at 5 h, and 0.0494 (*) at 6 h. Download FIG S5, TIF file, 0.07 MB. Copyright © 2021 Whittle et al. 2021 Whittle et al. https://creativecommons.org/licenses/by/4.0/ This content is distributed under the terms of the Creative Commons Attribution 4.0 International license.

10.1128/mBio.02608-21.6 FIG S6 Nile red accumulation in SL1344 and SL1344 Δ*tolC*. Median Nile red fluorescence per cell in 10,000 SYTO-9^+^ flow cytometry events was measured every hour between 1 and 6 h. Zero-hour time points were not included, as SYTO-9^+^ populations could not be gated. Single red triangles represent the X-median values of Nile red fluorescence in SL1344 (WT) (from 10,000 SYTO-9^+^ cell events) within a biological replicate. Individual orange triangles represent the X-median Nile red fluorescence in SL1344 Δ*tolC* (from 10,000 SYTO-9^+^ cell events) within a biological replicate. Four biological replicates for each strain are shown, with a short mean bar and error bars showing SEM. Nile red accumulation is plotted on the left *y* axis. Calculated cell numbers are plotted on the right *y* axis with corresponding symbols equating to strain and a dashed line to show growth of the culture. Cell numbers were based on the mean of the same biological replicates and the same gated population that Nile red fluorescence was measured from. Two-way ANOVA and Sidak’s multiple-comparison test were carried out for statistical analysis. ****, *P* < 0.0001. Download FIG S6, TIF file, 0.08 MB. Copyright © 2021 Whittle et al. 2021 Whittle et al. https://creativecommons.org/licenses/by/4.0/ This content is distributed under the terms of the Creative Commons Attribution 4.0 International license.

### Intracellular accumulation in stationary phase is controlled by reduced membrane permeability.

Together, these data show that cells from later growth phases minimize intracellular accumulation of EtBr (and other substrates) in an efflux-independent manner. We hypothesized this could be due to a shift in the balance between influx and efflux over growth, with influx rate, which is controlled by reduced permeability of the outer membrane, being more important in slower-growing or stationary-phase cells.

Several dyes that are often used to probe the permeability of the outer membrane, such as NPN (1-*N*-phenylnaphthylamine), are efflux substrates, and therefore, assessing membrane permeability in strains lacking efflux pumps is problematic. Most hydrophilic antibiotics enter Gram-negative bacterial cells through outer membrane porins such as OmpC and OmpF. To investigate whether porins altered the accumulation of EtBr, accumulation assays were performed using the mutants SL1344 Δ*ompC* Δ*ompF* Δ*acrB*, SL1344 Δ*ompC* Δ*acrB*, SL1344 Δ*ompF* Δ*acrB*, SL1344 Δ*ompC*, and SL1344 Δ*ompF* and showed that none had an EtBr accumulation pattern that was significantly different from those previously seen, confirming that EtBr does not enter *S*. Typhimurium through OmpF or OmpC (Fig. S7). A similar observation was made by Murata et al. in E. coli K-12 (32), and they concluded that the OM bilayer is the predominant mode of EtBr entry.

10.1128/mBio.02608-21.7 FIG S7 EtBr accumulation in strains of SL1344 and SL1344 Δ*acrB* with porin deletion. (A and B) Four biological replicates for each strain are shown, with a short mean bar and error bars showing SEM. EtBr accumulation is plotted on the left *y* axis. (A) SL1344 WT (individual green dots) versus SL1344 Δ*ompF* (green triangles), SL1344 Δ*ompC* (green squares), and SL1344 Δ*ompC* Δ*ompF* (blue diamonds). Median EtBr fluorescence per cell in 10,000 SYTO-84^+^ flow cytometry events was measured every hour between 0 and 6 h. Individual symbols represent the median value of EtBr fluorescence within a biological replicate. (B) SL1344 Δ*acrB* (red dots) versus SL1344 Δ*acrB* Δ*ompF* (red triangles), SL1344 Δ*acrB* Δ*ompC* (pink squares), and SL1344 Δ*acrB* Δ*ompC* Δ*ompF* (red diamonds). Median EtBr fluorescence per cell in 10,000 SYTO-84^+^ flow cytometry events was measured every hour between 0 and 6 h. Individual symbols represent the median value of EtBr fluorescence within a biological replicate. A two-way ANOVA and Dunnett’s multiple-comparison test were used for statistical analysis. Download FIG S7, TIF file, 0.1 MB. Copyright © 2021 Whittle et al. 2021 Whittle et al. https://creativecommons.org/licenses/by/4.0/ This content is distributed under the terms of the Creative Commons Attribution 4.0 International license.

**FIG 4:**
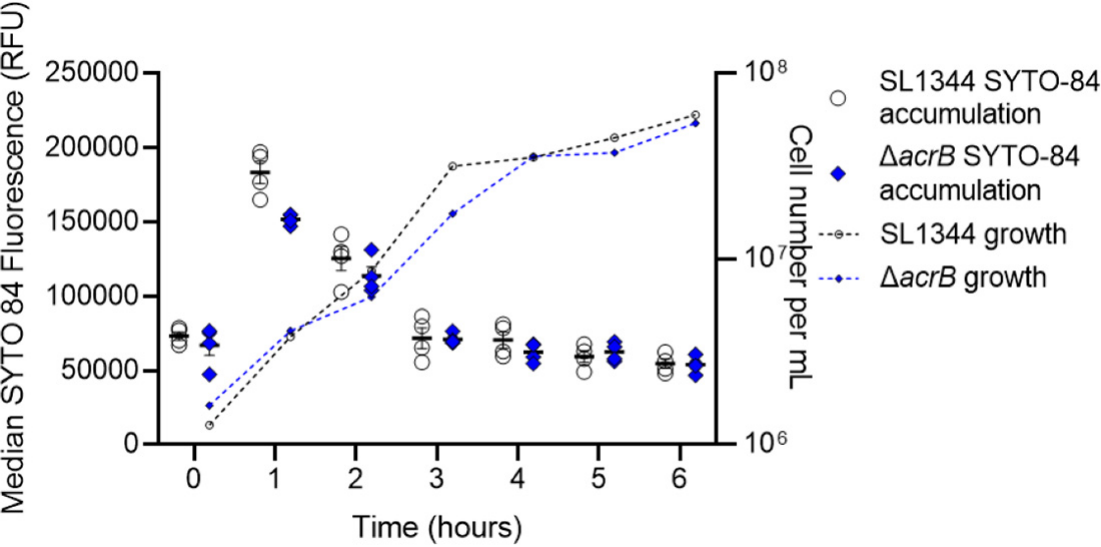
SYTO 84 accumulation in SL1344 and SL1344 Δ*acrB*. Median SYTO 84 fluorescence per cell in 10,000 cells was measured every hour between 0 and 6 h. White circles (SL1344) and blue diamonds (Δ*acrB*) represent the X-median value of SYTO 84 fluorescence in 10,000 cells within a biological replicate. Four biological replicates for each strain are shown; error bars show SEM. Median SYTO 84 fluorescence is plotted on the left *y* axis. Calculated cell numbers per milliliter are plotted on the right *y* axis with corresponding symbols equating to strain and a dashed line to show growth of the culture. Cell numbers were based on the mean of the same biological replicates and the same gated population that EtBr fluorescence was measured from.

Since SYTO 84 is used in our flow cytometry assay as a probe to stain cells, the accumulation of this dye was first investigated to assess permeability and to determine if it is an efflux substrate. SYTO 84 is marketed as a cell-permeant DNA dye and so is expected to readily enter bacteria. There was no significant difference between the accumulation of SYTO 84 in SL1344 and SL1344 Δ*acrB*, and in both strains, accumulation peaked after 1 h of growth (Fig. 4). This shows that SYTO 84 is not an efflux substrate and demonstrates the importance of efflux in maintaining low accumulation of drugs and dyes that are substrates in actively growing cells. However, SYTO 84 fluorescence decreased significantly in both strains on entrance to stationary phase (Fig. 4). This suggests that a compound that is not exported via efflux is also less able to enter bacteria during stationary phase, and we hypothesize that this is due to a strengthening of the permeability barrier. It is important to note that, although SYTO 84 fluorescence does decrease around 2.5-fold in stationary phase, the lowest value is still over 45,000 relative fluorescence units (RFU), so the reduction does not compromise its use to differentiate cells from acellular particles in the EtBr accumulation assays using flow cytometry.

**FIG 5:**
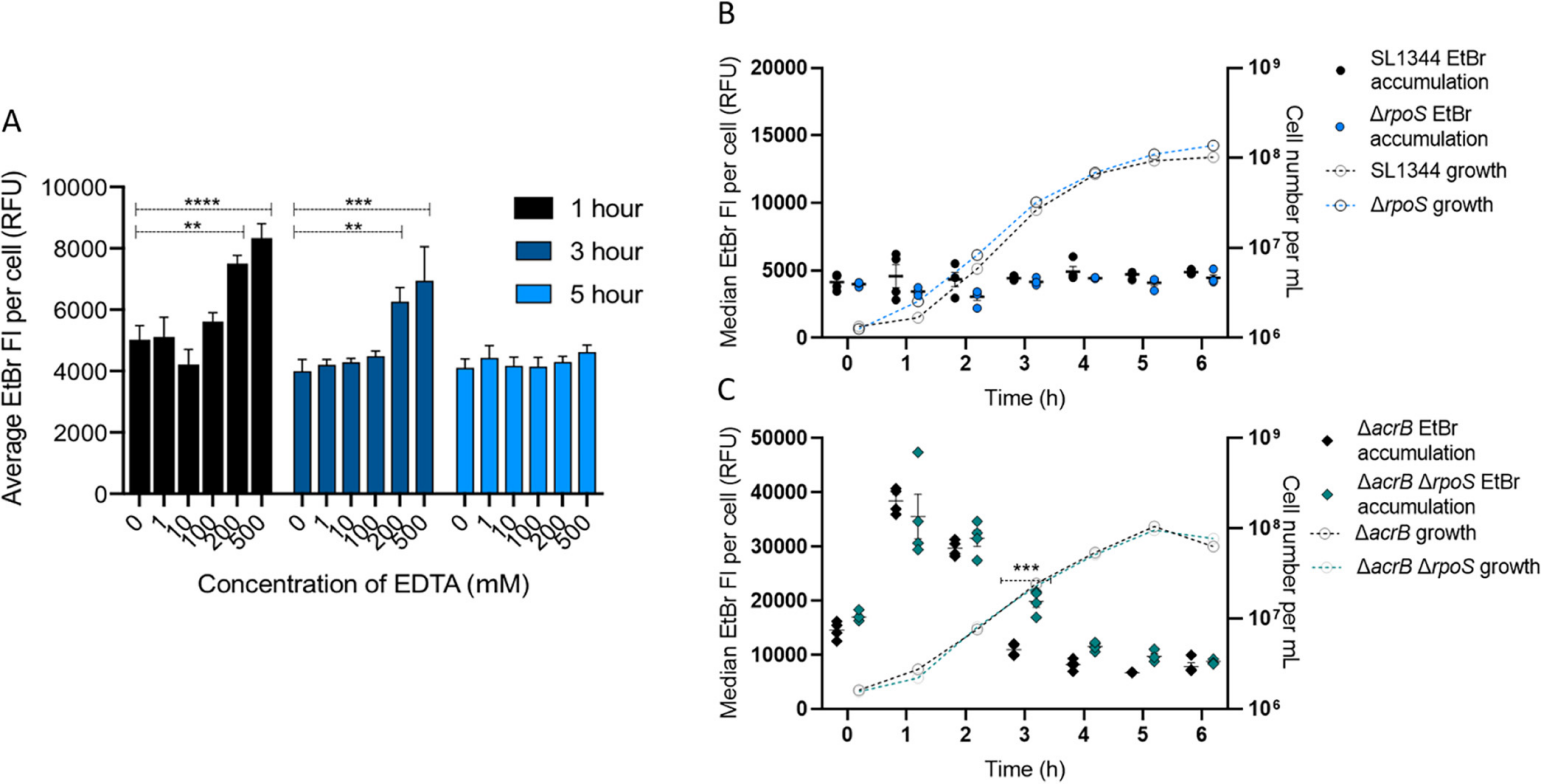
(A) EtBr accumulation in SL1344 treated with EDTA. Bars represent median EtBr fluorescence in 10,000 single cells of SL1344. EtBr accumulation was measured in the presence of increasing concentrations of EDTA (0, 1, 10, 100, 200, and 500 mM) from a culture grown for 1 h (black), 3 h (dark blue), and 5 h (light blue). Error bars show SEM from 3 biological replicates. Dashed lines above the bars with asterisks represent significance values based on a *t* test compared to the value when no EDTA was added. At 1 h, treatment with 200 and 500 mM significantly increased EtBr accumulation in SL1344 with *P* values of 0.0013 (**) and <0.0001 (****), respectively. At 3 h, treatment with 200 and 500 mM significantly increased EtBr accumulation in SL1344 with *P* values of 0.0033 (**) and 0.0001 (***), respectively. (B and C) EtBr accumulation in SL1344 Δ*rpoS* and SL1344 Δ*acrB* Δ*rpoS*. Four biological replicates for each strain are shown, with a short mean bar and error bars showing SEM. EtBr accumulation is plotted on the left *y* axis. Calculated cell numbers are plotted on the right *y* axis. Cell numbers were based on the mean of the same biological replicates and the same gated population that EtBr fluorescence was measured from. (B) SL1344 WT (individual black dots) versus Δ*rpoS* (blue dots). Median EtBr fluorescence per cell in 10,000 SYTO-84^+^ flow cytometry events was measured every hour between 0 and 6 h. Individual symbols represent the median value of EtBr fluorescence within a biological replicate. (C) SL1344 Δ*acrB* (black diamonds) versus SL1344 Δ*acrB* Δ*rpoS* (green diamonds). Median EtBr fluorescence per cell in 10,000 SYTO-84^+^ flow cytometry events was measured every hour between 0 and 6 h. Individual symbols represent the median value of EtBr fluorescence within a biological replicate. Significant differences to parent strain were measured by a two-way ANOVA and Sidak’s multiple-comparison test. At 3 h, EtBr accumulation in SL1344 Δ*acrB* Δ*rpoS* is significantly different from that in SL1344 Δ*acrB* with a *P* value of 0.0002 (***).

Ethidium bromide is a cationic dye that diffuses into cells through the OM (32). LPS molecules on the outer face of the outer membrane are ionically cross-linked to each other by divalent cations (Mg^2+^ or Ca^2+^) binding to phosphate groups in lipid A, generating a permeability barrier. EDTA is considered a permeabilizer which can chelate and thus displace divalent cations, destabilizing and releasing LPS (33), thereby increasing the permeability of the cell to itself and other compounds (6). Increasing concentrations of EDTA were used to permeabilize the outer membrane and assess the effect on ethidium bromide accumulation (Fig. 5A). Following 1 or 3 h of growth, there was no significant difference in EtBr accumulation up to 100 μM EDTA. At 200 μM and 500 μM EDTA, EtBr accumulation was significantly higher, suggesting that EDTA was able to make the outer membrane more permeable to EtBr. At 5 h, neither 200 μM nor 500 μM EDTA had any effect on the accumulation of EtBr. This suggests that the Salmonella outer membrane is remodeled during entry into stationary phase and becomes less reliant on cation-mediated cross-linking to maintain its permeability barrier to EtBr. Indeed, both Salmonella and E. coli become more resistant to CAMPs, whose mode of action relies upon interaction with negative charges on the LPS, in stationary phase (34, 35).

A previous study found that increased SDS resistance in carbon-limited stationary-phase E. coli is due to decreased envelope permeability mediated by RpoS-dependent and -independent mechanisms (19). The role of RpoS in decreased EtBr permeability in *S*. Typhimurium was therefore investigated by construction of Δ*rpoS* mutants of SL1344 and its Δ*acrB* variant.

Deletion of *rpoS* in SL1344 caused no significant difference in EtBr accumulation (Fig. 5B), although these bacteria were efflux active so EtBr could be pumped out. Comparison of the Δ*acrB* and Δ*rpoS* Δ*acrB* mutants (Fig. 5C) revealed a significant difference in EtBr accumulation only around 3 h growth; the Δ*rpoS* mutant showed a delayed decrease in EtBr accumulation, although in stationary phase, the two strains were similar. We conclude that in *S*. Typhimurium, although RpoS might play a role in envelope remodeling, it is not essential for generation of a low-permeability envelope in stationary phase, so there are likely to be RpoS-dependent and -independent pathways to achieve this phenotype. Although SDS and EDTA disrupt the cell envelope in different ways (detergent disruption of lipid membranes versus chelation of divalent cations), it is clear that RpoS-dependent and -independent mechanisms play a role in envelope remodeling in both E. coli (19) and *S*. Typhimurium.

### RNA-seq analysis identified several pathways likely to be involved in reduced envelope permeability in *S*. Typhimurium.

**FIG 6:**
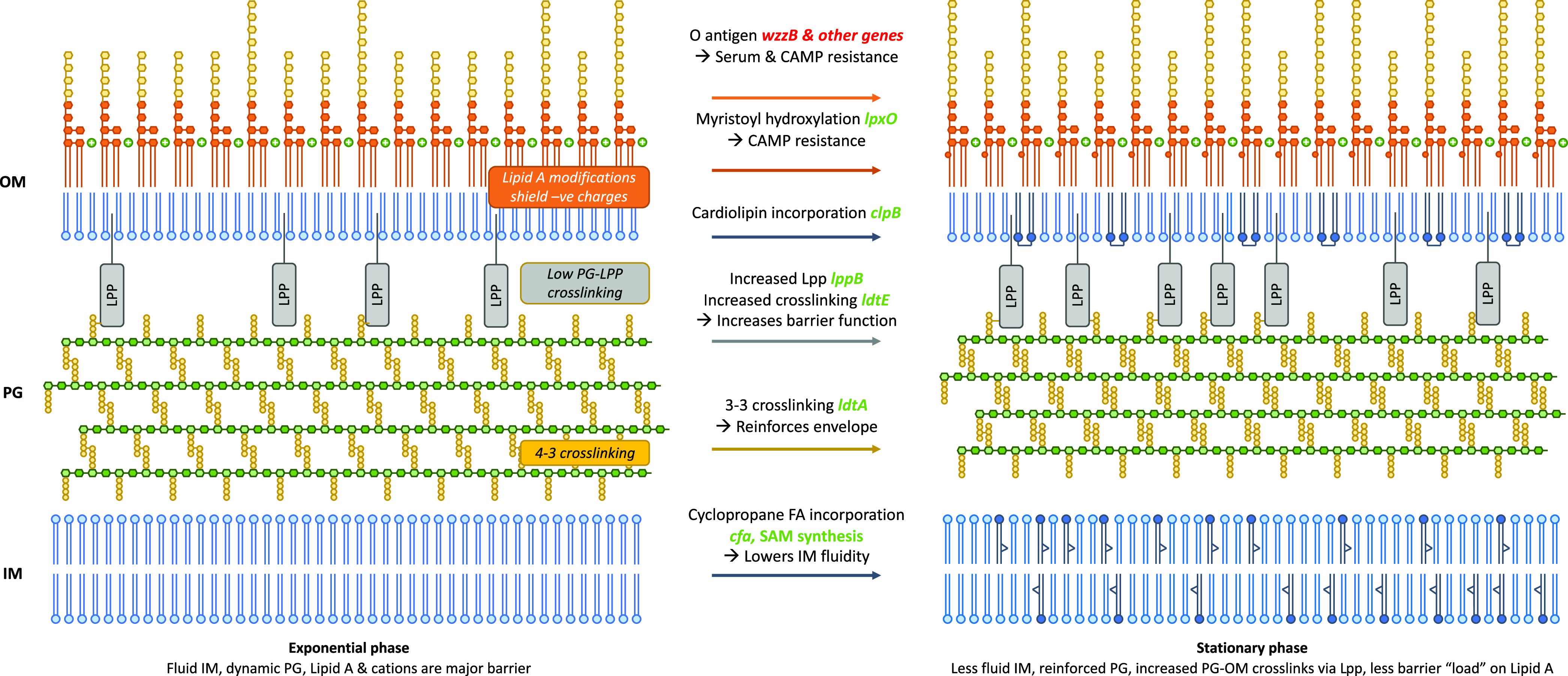
Model showing that the differentially expressed genes identified in the RNA-seq encode proteins involved in envelope remodeling in stationary phase to increase barrier function. Green text represents genes with increased expression, and red text represents genes with decreased expression.

Given that the data above did not identify a definitive mechanism by which the stationary-phase cell envelope displays lower permeability to EtBr, we used RNA-seq analysis to identify genes and pathways that may be involved in changes to Gram-negative cells as they enter stationary phase. Growing cultures of SL1344 were sampled after 1 h, 3 h, and 5 h of growth, and RNA was extracted and analyzed by GENEWIZ, Inc. Comparing SL1344 at 1 h versus 3 or 5 h of growth, 1,228 (26%) and 2,260 (47%) genes were differentially expressed, respectively. Differentially expressed genes were then identified that encode proteins involved in envelope remodeling in stationary phase, many of which have been shown to increase barrier function (Table S2; summarized in Fig. 6).

10.1128/mBio.02608-21.9 TABLE S2 Genes implicated in envelope remodeling that were identified by RNA-seq as being differentially expressed between 1 and 3 or 1 and 5 h growth. Fold changes are shown as 3 h/1 h or 5 h/1 h, so that a positive value indicates higher expression at 3 h or 5 h. Only genes with an adjusted *P* value of <0.05 and an absolute log_2_ fold change of >1 were considered differentially expressed. A dash signifies no significant difference in expression observed at that timepoint. Regulation data for E. coli (Ec) and Salmonella Typhimurium (ST) are taken from EcoCyc and the literature cited in the paper. Download Table S2, DOCX file, 0.02 MB. Copyright © 2021 Whittle et al. 2021 Whittle et al. https://creativecommons.org/licenses/by/4.0/ This content is distributed under the terms of the Creative Commons Attribution 4.0 International license.

Previous studies have suggested that multiple layers of the cell envelope are remodeled upon entry into stationary phase (34), and our RNA-seq data support this; a full description of this data set is in the supplemental material. Inner membrane fluidity decreases with cyclopropane fatty acid incorporation (36–38), mediated by upregulation of *cfa*. Stationary-phase peptidoglycan contains 3-3 (ld) rather than 4-3 (dd) cross-links (22, 39, 40); relevant transpeptidases are up- and downregulated. The quantity of Lpp in the OM increases (*lppB* is upregulated), and Lpp becomes more highly cross-linked to the PG (*ldtE* is upregulated [22]), which has been shown to increase barrier function (41). OM inner leaflet cardiolipin content is known to increase (42) potentially mediated by upregulation of *clsB*. LPS modification pathways important in exponential phase (e.g., the *pmr* genes, which confer CAMP resistance primarily through negative-charge neutralization) (43–45), are downregulated, whereas *lpxO* (involved in myristoyl chain hydroxylation and implicated in CAMP resistance in K. pneumoniae [46]) is upregulated. Genes involved in O-antigen synthesis and chain length regulation are downregulated; average O-antigen chain length increases in stationary phase, and O-antigen structure has been shown to influence serum resistance (47) and CAMP susceptibility (48). Finally, genes involved in enterobacterial common antigen (ECA) synthesis are downregulated; ECA is implicated in envelope integrity and bile resistance.

Taken together, these observations lead to a model (Fig. 6) suggesting why the exponential-phase cell envelope is more susceptible to attack from various factors (EtBr, CAMPs, EDTA, and antibiotics). Resistance to self-mediated uptake in exponential phase is provided primarily by the barrier function of LPS, comprising lipid hydrophobicity and cross-linking between phosphate groups and divalent cations. The LPS takes the burden because the inner layers of the envelope (peptidoglycan [PG] and the inner membrane [IM]) are by necessity more fluid; PG is extensively and continually remodeled to permit growth and division, and the IM is similarly fluid. The reliance on the LPS as the primary barrier poses problems when antimicrobials such as EDTA and CAMPs target the phosphate-cation bridges. The cell responds by shielding negative charges and modifying the LPS lipid content to decrease fluidity, regulated by PmrAB.

In stationary phase, each layer of the envelope plays a greater role in barrier function because remodeling and fluidity are less of a requirement. The lipid components of the IM and OM become less fluid, and the PG contains more ld cross-links and becomes more cross-linked to the OM, further strengthening the OM permeability barrier and decreasing (but not eliminating) the requirement for cation cross-linking of LPS. The saccharide components of the LPS provide the outermost layer of protection. This “laminated” approach shares the burden of protection and generates a strong barrier against multiple chemicals which seek to enter and damage the cell, reflected by the increased resistance of stationary-phase cells to multiple stressors.

## DISCUSSION

This study shows that the mechanisms that control drug accumulation are growth phase dependent. In actively growing cells, efflux is fundamental to maintaining low EtBr accumulation and subsequently survival of the bacterial population. Bacterial infections are complex, and bacterial populations will often not be in a single growth phase; therefore, careful consideration may be required for the most effective antibiotic treatment. We have shown that stationary-phase slow-growing or nongrowing cells are impermeable and that this is due not to changes in porin production but to membrane remodeling and increased peptidoglycan cross-linking, which reinforces the envelope barrier function.

The direct effect of these growth phase-dependent changes on susceptibility to antimicrobials is difficult to determine, as most MIC assays are growth based and take many hours, meaning that they are not sensitive enough to detect the effects of differences in intracellular antibiotic accumulation. In addition, they usually measure only one aspect of viability: the ability of bacteria to grow on an agar plate. This also means that bacteria with a viable-but-nonculturable (VBNC) phenotype cannot be detected. Future work will investigate antibiotic susceptibility in different growth phases in a non-growth-dependent manner.

However, our findings indicate that the treatment of chronic infections and biofilms where bacterial cells are slow growing or nongrowing may need to be considered more carefully. Successful treatment of these infections is already extremely difficult, and careful consideration is already made for treatment of intrinsically impermeable pathogens such as Pseudomonas aeruginosa and Acinetobacter baumannii. More extensive research into the effects of an impermeable membrane on treatment during infection must now be carried out.

Our findings also have implications for the development and use of efflux inhibitors. Efflux pumps are important in maintaining low accumulation only in actively growing cells, which have a more permeable envelope. If an infecting organism is actively growing, it seems likely that efflux inhibitors would be effective at increasing the accumulation of antibiotics within cells to potentiate their activity. However, if cells are in a slow-growing or nongrowing state, where membrane permeability is fundamental to maintaining low drug accumulation, efflux inhibitors may not be an effective treatment option. It is also possible that administering an efflux inhibitor where it has no effect on treating an infection may also lead to the development of new mechanisms of antimicrobial resistance.

## MATERIALS AND METHODS

### Strains and growth conditions.

Unless otherwise stated, all experiments used Salmonella enterica serovar Typhimurium SL1344 (49). The Δ*acrB* and Δ4PAP (Δ*acrA* Δ*acrE* Δ*mdsA* Δ*mdtA*) strains were described previously (50, 51). SL1344 Δ*ompF* and Δ*ompC* strains were constructed for this study using the Datsenko and Wanner method of gene deletion (52). Transcriptional reporter constructs were made by fusing the promoter of each efflux pump gene to the GFP gene in the pMW82 plasmid (53). These plasmids were transformed into SL1344 and SL1344 Δ*acrB*. E. coli MG1655 Δ*acrB* (15), P. aeruginosa PAO1 Δ*mexA* (54), and K. pneumoniae ecl8 *acrB*::Gm (17) were also used as part of this study and were described previously. All oligonucleotides are listed in Table S1.

10.1128/mBio.02608-21.8 TABLE S1 Oligonucleotides. Download Table S1, DOCX file, 0.01 MB. Copyright © 2021 Whittle et al. 2021 Whittle et al. https://creativecommons.org/licenses/by/4.0/ This content is distributed under the terms of the Creative Commons Attribution 4.0 International license.

Unless otherwise stated, LB (Sigma) was used as the growth medium for all assays. One assay used MOPS minimal medium (Teknova), which was supplemented with 400 mg/liter histidine.

### Chromosomal insertion of *gfp* downstream of *acrB* to produce SL1344 AcrB-GFP.

To measure the protein level of AcrB in *S*. Typhimurium, a gene encoding a monomeric superfolder GFP (msfGFP) was inserted downstream of *acrB* on the chromosome to produce an AcrB-msfGFP fusion protein. This strain was created using the msfGFP from the pET GFP LIC cloning vector (u-msfGFP), which was a gift from Scott Gradia (Addgene plasmid number 29772; http://n2t.net/addgene:29772; RRID, Addgene_29772). Strain construction was based on the method used by Bergmiller et al. in E. coli (27) where the codon-optimized polylinker was used. Using restriction and ligation, the *aph* gene was inserted into pET LIC vector (u-msfGFP), so that strains containing the plasmid could be selected for. Using this plasmid as the template, *gfp* and *aph* were inserted into the chromosome downstream of *acrB* in SL1344 to produce a protein fusion strain.

### Flow cytometry assay.

The flow-cytometric EtBr accumulation assay was described previously (17). Here, this method was used to measure accumulation in samples from the same culture at different time points during batch culture. Briefly, cultures were grown at 37°C overnight in 5 ml of LB and subcultured at 4% into fresh LB. A sample was taken at 0 h and then every hour for 6 h during growth. At each hour, sample volume was adjusted such that approximately 10^7^ cells were harvested and resuspended in 1× HEPES-buffered saline (5× HBS; Alfa Aesar). Cells were washed and resuspended in 1 ml HBS. One hundred microliters of cell suspension was then further diluted into 500 μl HBS and SYTO 84 (Thermo Fisher Scientific) and ethidium bromide added to give final concentrations of 10 μM and 100 μM, respectively. Samples were incubated for 10 min before measuring accumulation by flow cytometry. Flow cytometry settings and emission filters described by Whittle et al. (17) were used. Briefly, the SYTO 84 fluorescence emission was collected in the YL1-H channel (585/16 nm) using a 561-nm yellow laser and used to differentiate cells from acellular material. EtBr fluorescence was collected using the BL3-H channel (695/40 nm) using a 488-nm blue laser. SYTO 84 accumulation measurements (Fig. 4) were not obtained in a repeated experiment, but data were reanalyzed from EtBr accumulation assays, and therefore fluorescence emission was collected in the YL1-H channel (585/16 nm) using a 561-nm yellow laser. Nile red accumulation was measured as previously described (17). In these experiments SYTO 9 (10 μM; Thermo Fisher Scientific) was used to differentiate cells from acellular particles using the BL2-H channel. Nile red has an excitation wavelength of 549 nm and an emission wavelength of 628 nm in the presence of phospholipids, and in a neutral lipid environment (triglycerides), the fluorescence shifts to excitation/emission of 510/580 nm (31). Nile red fluorescence was excited using the yellow laser and detected using the YL1-H channel for orange fluorescence (17).

### Flow cytometry assay in the presence of EDTA.

Growing culture samples were taken at 1, 3, and 5 h as described above. Samples were made with various concentrations of EDTA (0 μM, 1 μM, 10 μM, 100 μM, 200 μM, and 500 μM) in 500 μl HBS. These concentrations of EDTA increased the final volume of the sample because the stock concentration was limited by solubility. Dyes were then added, but the volume added was adjusted to maintain the final concentrations stated above. Once the dyes were added, 100 μl of cell suspension was added and cells were incubated for 10 min at room temperature. Samples were then analyzed by flow cytometry.

### Whole-population transcription analysis.

Overnight cultures containing pMW82 transcriptional reporter plasmids were diluted 1:10,000 in MOPS minimal medium, supplemented with 50 μg/ml ampicillin. Optical density at 600 nm (OD_600_) and GFP fluorescence were measured every 30 min for 12 h using a Fluostar Omega (BMG Labtech) incubated at 37°C. OD_600_ and GFP fluorescence were measured, and values for a minimal-medium-only control were subtracted from the data. SL1344 autofluorescence was removed by subtracting SL1344 fluorescence from that of pMW82 strains. GFP fluorescence divided by OD_600_ was used as a measurement to disregard cell density across growth.

### Efflux assay.

Efflux assays were carried out as previously described (14). This assay measures direct efflux activity of a population of cells by preloading cells with a fluorescent efflux substrate in the presence of the proton motive force inhibitor, CCCP, and re-energizing cells with glucose to measure the decrease in fluorescence as substrates leave the cells. Briefly, overnight cultures of SL1344 and SL1344 Δ*acrB* were subcultured into fresh LB and then grown for 5 h at 37°C. At 1, 3, and 5 h, 10 ml of culture was taken and the OD_600_ measured. The harvested cell pellet was then resuspended in phosphate buffer containing MgCl_2_ buffer, and each strain was adjusted to the same OD_600_.

### RNA-seq.

The transcriptomes of SL1344 and SL1344 Δ*acrB* were analyzed at different time points during growth (1, 3, and 5 h). There were 4 replicates of each strain. MOPS minimal medium was inoculated at 4% with overnight cultures. Cultures were incubated at 37°C with shaking for 5 h. At 1 h, 5 ml of culture was centrifuged at 3,500 × *g* for 5 min at room temperature to harvest the cells. The supernatant was removed and the pellet was snap-frozen. At 3 and 5 h, only 1 ml of culture was harvested and snap-frozen. GENEWIZ, Inc., carried out the RNA extraction, quality control, library preparation, sequencing, and bioinformatic analysis. Briefly, total RNA was extracted from *S*. Typhimurium cell pellets using an RNeasy Plus universal kit (Qiagen), and RNA quality control was carried out using a Qubit 2.0 fluorometer to measure total RNA concentration and an Agilent TapeStation to produce an RNA integrity number (RIN) and a DV200 score. To remove rRNA, the Ribozero removal kit was used (Illumina). The NEBNext Ultra II RNA library preparation kit (Illumina) was used for library preparation, following the manufacturer’s protocol. For library preparation, cDNA was synthesized, end repaired, and adenylated at the 3′ ends. Universal adapters were ligated to cDNA, and library enrichment was carried out using limited-cycle PCR. Sequencing was carried out using Illumina HiSeq 4000. Bioinformatic data analysis was carried out by GENEWIZ, Inc. Trimmed reads were mapped to the SL1344 reference genome FQ312003 using the Bowtie2 aligner. Unique gene hit counts were calculated by using featureCounts from the Subread package. All statistical analysis was performed using R. With the package DESeq2, a comparison of gene expression between the groups of samples was performed. The Wald test was used to generate *P* values and log_2_ fold changes.

### Data availability.

The data have been deposited with ArrayExpress (accession no. E-MTAB-9679).

10.1128/mBio.02608-21.10 TEXT S1 Supplemental methods and discussion. Download Text S1, DOCX file, 0.07 MB. Copyright © 2021 Whittle et al. 2021 Whittle et al. https://creativecommons.org/licenses/by/4.0/ This content is distributed under the terms of the Creative Commons Attribution 4.0 International license.
