# Supplementary figures and images for: Efflux Impacts Intracellular Accumulation Only in Actively Growing Bacterial Cells
Source: mBio. 2021 Oct 12;12(5):e02608-21. doi: 10.1128/mBio.02608-21 (PMC8510537; doi:10.1128/mBio.02608-21)

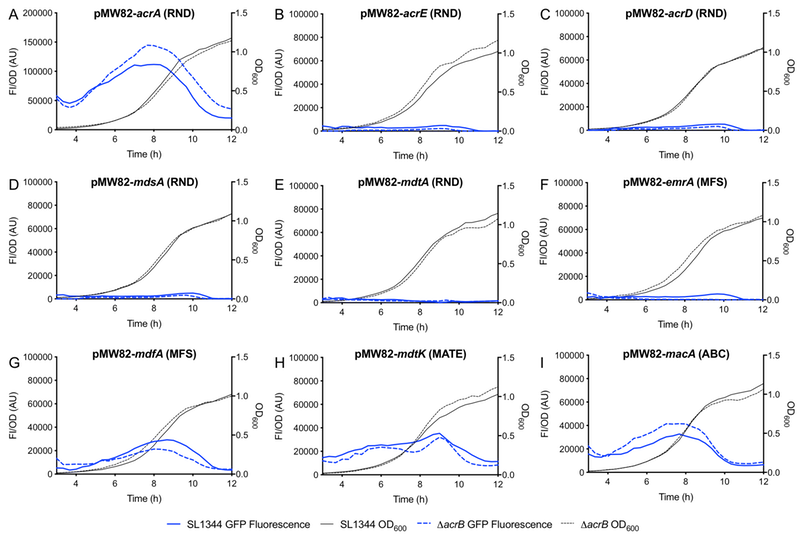

Supplement: FIG S1 [file mbio.02608-21-sf001.tif]

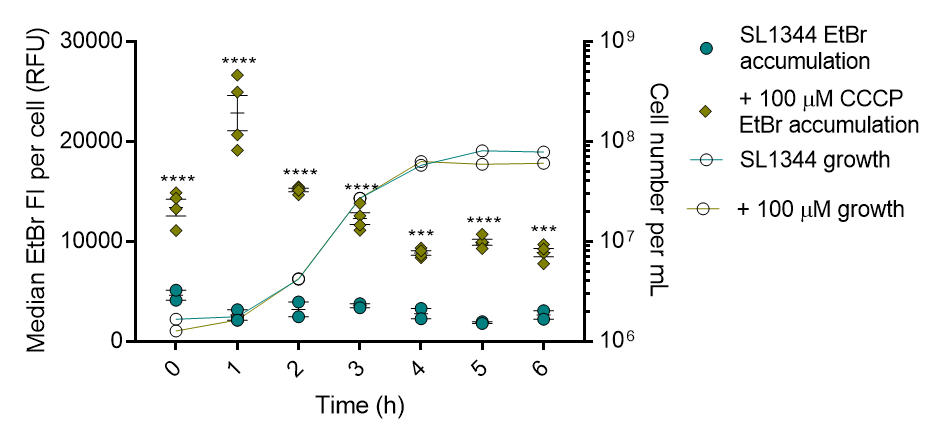

Supplement: FIG S2 [file mbio.02608-21-sf002.tif]

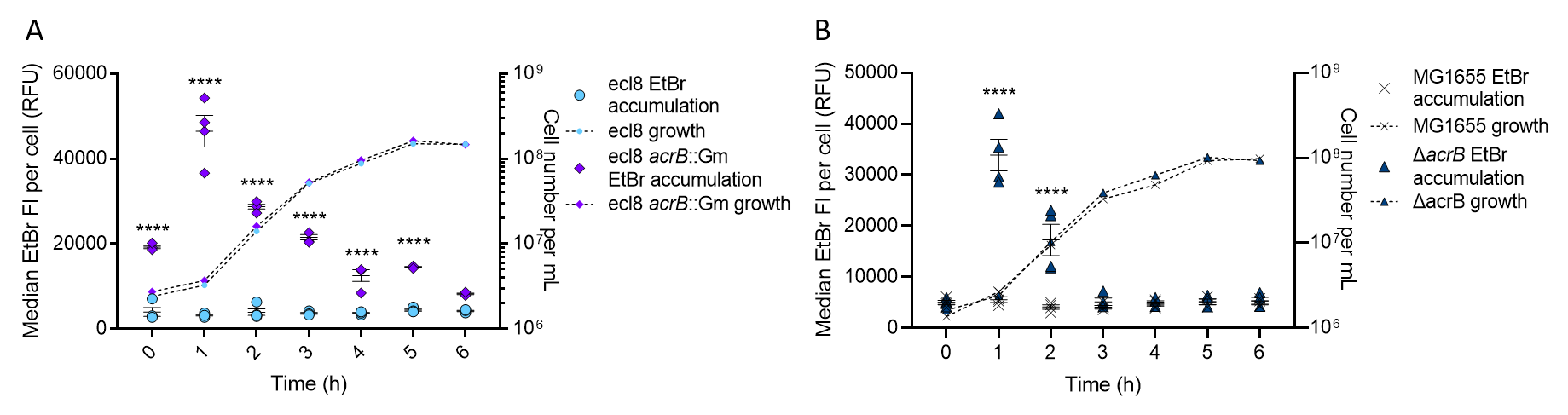

Supplement: FIG S3 [file mbio.02608-21-sf003.tif]

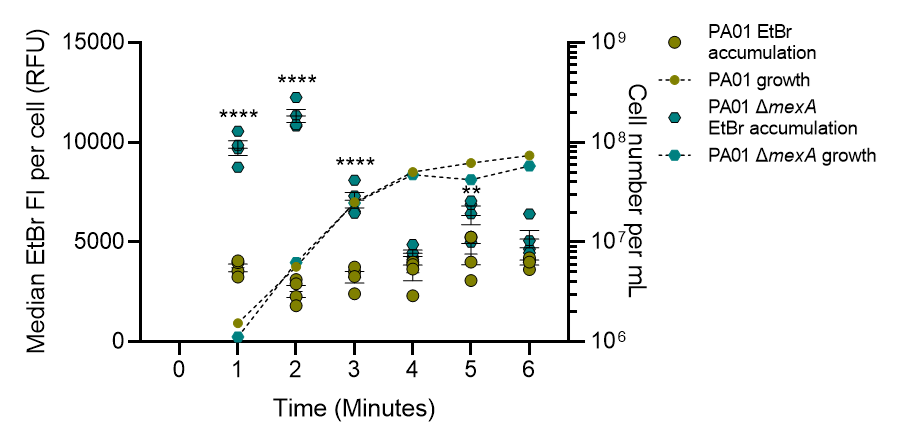

Supplement: FIG S4 [file mbio.02608-21-sf004.tif]

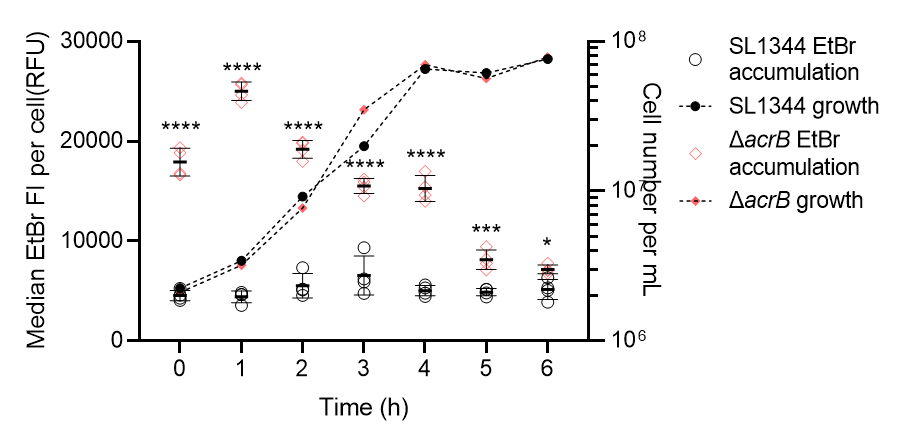

Supplement: FIG S5 [file mbio.02608-21-sf005.tif]

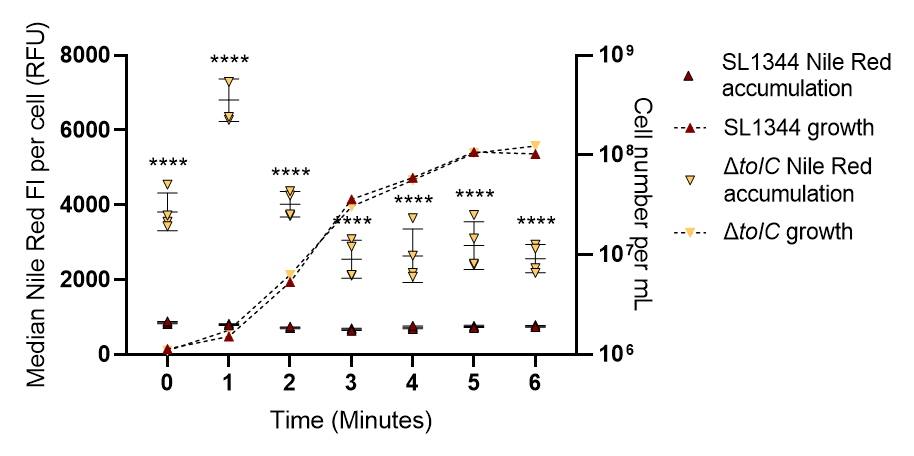

Supplement: FIG S6 [file mbio.02608-21-sf006.tif]

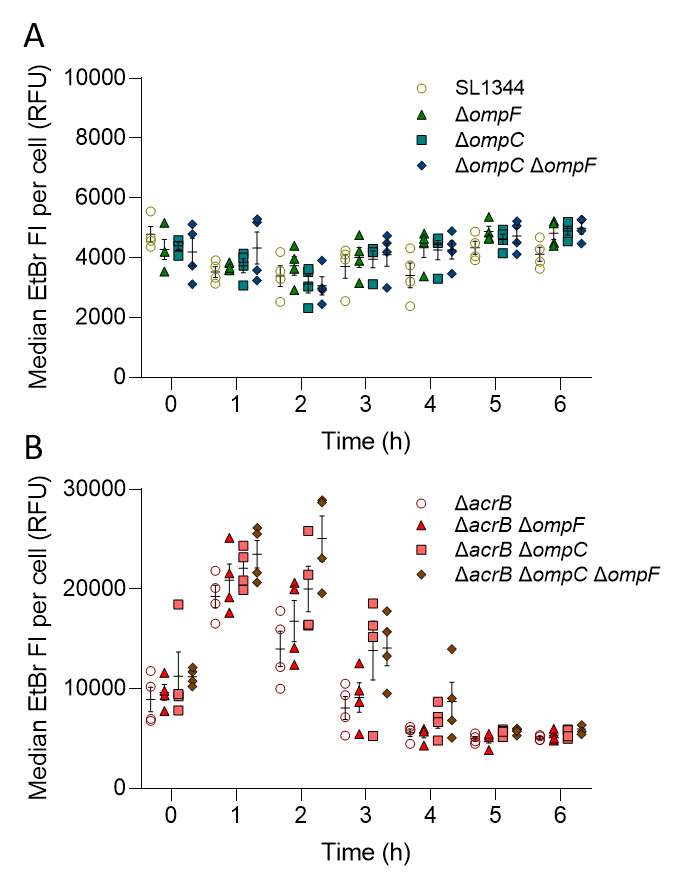

Supplement: FIG S7 [file mbio.02608-21-sf007.tif]
