# Supplementary material for: Efflux Impacts Intracellular Accumulation Only in Actively Growing Bacterial Cells
Source: mBio. 2021 Oct 12;12(5):e02608-21. doi: 10.1128/mBio.02608-21 (PMC8510537; doi:10.1128/mBio.02608-21)
Supplement: TABLE S2 [file mbio.02608-21-st002.docx]

**Table S2.** Genes implicated in envelope remodeling that were identified by RNASeq as being differentially expressed between 1 and 3 or 1 and 5 hours growth.

| **Pathways** | **Function** | | **gene** | **1 vs 3 hr fold change** | **1 vs 5 hr fold change** | **Regulation** |
| --- | --- | --- | --- | --- | --- | --- |
| CFA biosynthesis | Conversion of unsaturated fatty acids to cyclopropane fatty acids | | *cfa* | 3.17 | 7.27 | Ec – RpoS and ppGpp activated, sRNA regulated |
| L-methionine and SAM biosynthesis | Synthesis of the methyl donor SAM | | *metJ* | 4.29 | 2.08 |  |
|  |  |  | *metQ* | 4.33 | 2.30 | Ec – MetJ repressed |
|  |  |  | *metC* | 10.71 | 4.15 | Ec – MetJ repressed |
|  |  |  | *metK* | 11.02 | 6.20 | Ec – MetJ repressed |
|  |  |  | *metI* | 12.00 | 4.36 | Ec – MetJ repressed |
|  |  |  | *metN* | 17.43 | 3.79 | Ec – MetJ repressed |
|  |  |  | *metL* | 27.63 | 3.20 | Ec – MetJ repressed, PhoP activated |
|  |  |  | *metA* | 42.82 | 13.61 | Ec – MetJ repressed |
|  |  |  | *metB* | 48.08 | 2.21 | Ec – MetJ repressed, PhoP activated |
|  |  |  | *metR* | 51.96 | 5.54 | Ec – MetJ, MetR repressed |
|  |  |  | *metF* | 155.57 | 37.62 | Ec – MetJ repressed |
|  |  |  | *metE* | 236.20 | 64.66 | Ec – MetJ repressed, MetR activated |
| Peptidoglycan biosynthesis and remodelling | glycosyltransferases | | *mrcA* | 0.40 | 0.29 |  |
|  |  |  | *mtgA* | 2.41 | 2.88 |  |
|  | D,D‑carboxypeptidases | PG remodelling | *mrcA* | 0.40 | 0.29 |  |
|  |  |  | *dacA* | 0.46 | 0.19 |  |
|  |  |  | *dacC* | - | 2.34 | Ec – ppGpp activated |
|  | D,D‑transpeptidases | Forms 4-3 PGcrosslinks | *mrcA* | 0.40 | 0.29 |  |
|  |  |  | *mrdA* | 0.47 | 0.29 |  |
|  |  |  | *ftsI* | 0.29 | 0.45 |  |
|  | L,D‑transpeptidases | Crosslinks PG to Lpp | *ldtA* | - | 2.50 | Ec – RpoS activated |
|  |  |  | *ldtB* | 0.12 | 0.10 |  |
|  |  | Forms 3-3 PG crosslinks | *ldtE* | 2.44 | 2.96 | Ec – RpoS and ppGpp activated |
|  | Amidases | PG remodelling | *amiC* | 0.49 | 0.46 |  |
|  |  |  | *amiD (ybjR)* | - | 2.41 |  |
|  | Lytic transglycosylases | PG remodelling | *mltA* | - | 0.26 |  |
|  |  |  | *mltC* | - | 0.35 |  |
|  |  |  | *mltD* | 0.30 | 0.11 |  |
|  |  |  | *mltF* | 0.38 | 0.39 |  |
|  | D,D‑endopeptidases | PG turnover and remodelling | *mepS* | 0.31 | 0.25 |  |
|  |  |  | *mepM* | 0.31 | 0.26 |  |
|  |  |  | *pbpG* | 0.36 | 0.36 |  |
| Braun lipoprotein | Links OM to PG | | *lppB* |  | 2.10 |  |
| Cardiolipin synthesis | Synthesis of cardiolipin from phosphatidylglycerol | | *clpB* |  | 2.61 | Ec - ppGpp activated |
| Lipid A biosynthesis | Synthesis of lipid A component of LPS | | *lpxH* | 0.28 | 0.17 |  |
|  |  |  | *lpxB* | 0.41 | 0.21 |  |
|  |  |  | *lpxK* | 0.38 | 0.21 |  |
|  |  |  | *lpxA* | - | 0.25 |  |
|  |  |  | *lpxL* | - | 0.30 |  |
|  |  |  | *lpxD* | - | 0.41 |  |
| Lipid A modifications | Two‑component regulatory system, regulator of polymyxin resistance | | *pmrA* | - | 0.18 |  |
|  |  |  | *pmrB* | 0.49 | 0.11 |  |
|  | Addition of 4-amino-4-deoxy-L-arabinose (L‑Ara4N) to lipid A | | *arnA (pmrI)* | - | 0.07 | ST- PmrAB activated |
|  |  |  | *arnB (pmrH)* | - | 0.06 | ST- PmrAB activated |
|  |  |  | *arnC (pmrF)* | - | 0.09 | ST- PmrAB activated |
|  |  |  | *udg (pmrE)* | - | 0.10 | ST- PmrAB activated |
|  |  |  | *arnT (pmrK)* | 0.46 | - | ST- PmrAB activated |
|  | Myristoyl chain hydroxylation | | *lpxO* | - | 2.54 | ST- PhoPQ activated |
|  | Addition of ethanolamine to lipid A | | *eptA (pmrC)* | - | 0.06 | ST- PmrAB activated |
|  | Addition of palmitate to lipid A and phospholipids | | *pagP* | 0.28 | 0.11 | Ec – PhoP activated |
|  | Palmitoleote Incorporation | | *lpxP* | 0.13 | 0.05 |  |
| LPS core modifications | Adds phosphoethanolamine to heptose (I) phosphate in LPS core | | *cptA* | - | 0.19 | ST- PmrAB activated |
|  | Dephosphorylates heptose (II) phosphate in LPS core | | *pmrG (ais)* | - | 0.08 | ST - PmrAB activated |
| O antigen synthesis and modification | O-antigen acyltransferase | | *oafA* | - | 0.08 |  |
|  | O-antigen translocase | | *wzx (rfbX)* | - | 0.49 |  |
|  | O-antigen polymerase | | *wzy (rfc)* | - | 0.39 |  |
|  | O antigen biosynthesis rhamnosyltransferase | | *wbaN (rfbN)* | - | 0.35 |  |
|  | O-antigen chain length determinant protein Wzz_ST_ | | *wzzB* | 0.39 | 0.18 | ST- PmrAB activated |
|  | O-antigen ligase | | *waaL (rfaL)* | 0.28 | 0.16 |  |
| ECA subunit biosynthesis | UDP-N-acetyl-D-mannosamine dehydrogenase | | *wecC* | - | 0.40 |  |
|  | dTDP-glucose 4,6-dehydratase | | *rffG* |  | 0.43 |  |
|  | dTDP-fucosamine acetyltransferase | | *wecD* | - | 0.39 |  |
|  | Lipid III flippase | | *wzxE* | 0.45 | 0.34 |  |
|  | TDP-N-acetylfucosamine:lipid II N-acetylfucosaminyltransferase | | *wecF* | 0.39 | 0.21 |  |
|  | UDP-N-acetyl-D-mannosaminuronic acid transferase | | *wecG* | - | 0.47 |  |

Fold changes are shown as 3h/1h or 5h/1h so that a positive value indicates higher expression at 3h or 5h. Only genes with an adjusted p value of <0.05 and an absolute log2 fold change >1 were considered as differentially expressed. A dash signifies no significant difference in expression observed at that timepoint. Regulation data for *E. coli (*Ec) and *Salmonella* Typhimurium (ST) are taken from Ecocyc and the literature cited in the manuscript.
