# Supplementary material for: Efflux Impacts Intracellular Accumulation Only in Actively Growing Bacterial Cells
Source: mBio. 2021 Oct 12;12(5):e02608-21. doi: 10.1128/mBio.02608-21 (PMC8510537; doi:10.1128/mBio.02608-21)
Supplement: TABLE S1 [file mbio.02608-21-st001.docx]

| Table S1. List of oligonucleotides | | |
| --- | --- | --- |
| Code | **Description** | **Sequence (5’-3’)** |
| 242 | (F) for inserting *aph* into msfGFP with upstream AscI site | TATATTGGCGCGCCGTGTAGGCTGGAGCTGCTTC |
| 243 | (R) for inserting *aph* into msfGFP with downstream KpnI site | AGGATATTCATATGGACCATGGCTAATTCCCATGGTACCCCGATA |
| 246 | *aph* insertion into msfGFP (F) | GTCCAAGCTGAGCAAAGACC |
| 247 | *aph* insertion into msfGFP (R) | TGAATGAACTGCAGGACGAG |
| 261 | (F) to insert msfGFP-*aph* downstream of *acrB* | GAGCATAGTCATTCGACAGAACATCGCGGTAGCGGTAACAAAGGTCAGGGCGTGAGCAAGGGCGAGGAGCTGTT |
| 249 | (R) for the insertion of msfGFP-*aph* downstream of *acrB* | GGACCATGGCTAATTCCCATTTTGCTCACTGTTGATAAGGCCGCGCAAGCGGCCTTTTTTACGCAAAAATCT |
