# Supplementary material for: Efflux Impacts Intracellular Accumulation Only in Actively Growing Bacterial Cells
Source: mBio. 2021 Oct 12;12(5):e02608-21. doi: 10.1128/mBio.02608-21 (PMC8510537; doi:10.1128/mBio.02608-21)
Supplement: TEXT S1 [file mbio.02608-21-s0001.docx]

**Supplementary Material: Efflux only impacts drug accumulation in actively growing bacterial cells**

**Emily E Whittle^1^, Helen E McNeil^1^, Eleftheria Trampari^2^, Mark Webber^2^, Tim W Overton^3^, Jessica M A Blair^1*^**

**Supplementary methods**

**Chromosomal insertion of *gfp* downstream of *acrB* to produce SL1344 AcrB-GFP**

To measure the protein level of AcrB in *S.* Typhimurium, a gene encoding a monomeric super‑folder GFP (msfGFP) was inserted downstream of *acrB* on the chromosome to produce a AcrB-msfGFP fusion protein. This strain was created using the msfGFP from the pET GFP LIC cloning vector (u-msfGFP) was a gift from Scott Gradia (Addgene plasmid # 29772 ; http://n2t.net/addgene:29772 ; RRID:Addgene_29772). Strain construction was based on the method used by Bergmiller et al. (2017) in *E. coli* (1) where the codon optimised polylinker was used*.* Using restriction and ligation, the *aph* gene was inserted into pET LIC vector (u‑msfGFP), so that strains containing the plasmid could be selected for. Using this plasmid as template, *gfp* and *aph* were inserted into the chromosome downstream of *acrB* in SL1344 to produce a protein fusion strain.

The restriction sites downstream of the *gfp* gene that enable cutting by AscI and KpnI‑HF (NEB) restriction enzymes, were used to insert *aph* into the msfGFP encoding plasmid. The *aph* gene was amplified from the pKD4 plasmid. The forward oligonucleotide contained the AscI restriction site at the 5’ end, where the KpnI restriction site was present at the 5’ end of the reverse oligonucleotide. Restriction digest was carried out on the msfGFP plasmid and the *aph* DNA product using the enzymes stated above to produce overhangs of DNA for re‑ligation. The ligation reactions were then transformed in to NEB 5‑ α *E. coli* (C29871) using the NEB transformation protocol.

The msfGFP + *aph* plasmid was purified using the Qiagen plasmid prep protocol. Oligonucleotides were designed to amplify GFP and *aph* from the plasmid with homology to *acrB*. The forward oligonucleotide shown in **Table S1**, was designed to have homology to the 3’ end of *acrB,* a codon optimised polylinker (GgtAgcGgtAacAaaGgtCagGgc) (1) and homology to the 5’ of *gfp.*The reverse oligonucleotide had homology to the end of *aph* and a non‑coding downstream region of *acrB*. Insertion of *gfp* and *aph* downstream of *acrB* was done using homologous recombination (2),. PCR and sequencing confirmed correct fusion of *gfp* to *acrB* and then *aph* was removed using pCP20 (2, 3).

**Supplementary Discussion**

**Evidence for envelope remodeling from RNAseq**

Starting at the inner membrane, expression of *cfa* had significantly increased expression at 3 or 5 hours of growth compared to 1 hour in SL1344 (3.17-fold change and 7.27-fold change respectively). The *cfa* gene product converts linear unsaturated fatty acids (attached to phospholipids) to cyclopropane fatty acids via the addition of a methyl group donated by *S*-adenosylmethionine (SAM). It should also be noted that genes encoding the SAM synthesis pathway are highly upregulated at 3 and 5 hours of growth. It has previously been shown that cyclopropane fatty acids accumulate in the inner membrane in stationary phase, with the onset of starvation, and have been hypothesised to lead to a decrease in membrane fluidity (4–6). It has also been shown that the *cfa* gene is in part regulated by RpoS (7, 8) Mutants lacking *cfa* display increased susceptibility to acid, heat and pressure in stationary phase, further highlighting the importance of cyclopropane fatty acids in tolerating these stresses via membrane mediated mechanisms (9, 10). It is therefore likely that increased *cfa* expression in stationary phase results in decreased inner membrane fluidity and enhanced barrier function.

***Peptidoglycan***

A key component of the Gram-negative envelope is the peptidoglycan (PG) cell wall. It has previously been shown that in stationary phase PG makes up a larger proportion of dry cell weight (11) and is more highly crosslinked (12). Transpeptidase enzymes that introduce crosslinks between PG peptide chains were differentially expressed in our RNAseq dataset. In *E. coli*, the majority of peptidoglycan crosslinks are 4-3 or DD crosslinks between d-Ala and meso-diaminopimelic acid (mDAP). Of the 4 DD-transpeptidases, *mrcA (*PBP1A), *mrdA (*PBP2) and *ftsI* (PBP3) had decreased expression at 5 hours, and *mrcB* (PBP1B) remained unchanged. A smaller percentage of crosslinks occur between mDAP and mDAP; the number of these 3-3 or LD crosslinks increase dramatically in stationary phase (12–14). Of the two LD-transpeptidases (LDTs) that form 3-3 crosslinks, transcription of *ldtD* was unchanged at 5 hours of growth compared to 1 hour in our dataset, but *ldtE* (*ynhG*) expression increased. In *E. coli*, *ldtE* has been described as the ‘housekeeping’ stationary phase LDT (13); its expression is activated by RpoS and ppGpp (15, 16). It is proposed that 3-3 crosslinks reinforce the envelope in non-replicating conditions and in response to stress (17, 18). In clinical isolates, bacteria with increased LD crosslinks can be highly resistant to β-lactams because they do not require PBPs for transpeptidation (19).

In *E. coli* three further LDTs (LdtA, LdtB and LdtC) have been shown to crosslink the PG to Braun lipoprotein Lpp, which links the PG to the OM and thus regulates the width of the periplasm (20, 21). The proportion of Lpp crosslinked in this manner increases in stationary phase (12). In our dataset, *ldtA* was upregulated at 5 h while *ldtB* was downregulated at 3 and 5 hours. In *E. coli*, *ldtA* expression is RpoS-dependent (15). A triple *E. coli* *ldtA ldtB ldtC* knockout leaks periplasmic proteins and is sensitive to EDTA, as is an *lpp* mutant, although a double *ldtD ldtE* knockout is not (17). It is therefore suggested that the PG-OM linkage via Lpp is critical for OM stability and barrier function (19). It is also worth noting that *lppB* expression increased at 5 h in our dataset; *S.* Typhimurium has two genes encoding Lpp, *lppA* and *lppB*.

Two genes encoding glycosyltransferases that polymerise PG from Lipid II subunits were oppositely regulated in our dataset; *mrcA* (PBP1A) was downregulated and *mtgA* upregulated at 3 and 5h. We believe that this observation is novel.

Expression of PG hydrolases was also seen to change in our RNASeq data; this class of enzymes is essential for PG turnover and remodelling, cell growth, and other functions (22). Three DD-endopeptidases (MepS, MepM and PbpG) which break crosslinks between d-Ala and mDAP, had decreased expression at 3h and 5h. These endopeptidases are required for PG remodeling to permit cell elongation during exponential growth (23).

Four genes encoding lytic transglycosylases (*mltA, mltC, mltD*, and *mltF*) that cleave bonds between the sugar residues are downregulated at 5 h. This class of enzymes are important for multiple functions including PG remodeling during growth (24, 25), so it is logical that they are downregulated in stationary phase. It has been suggested that their contribution to cleavage and cell division is reduced in comparison to amidases in *E. coli* (26).

The DD-carboxyopetidases *dacA* (PBP5) and *dacC* (PBP6) which cleave the terminal d-Ala from PG peptide chains were observed to be down- and up-regulated at 5 h in our dataset. Expression of *dacC* is induced in stationary phase and is ppGpp activated (16). DacC is postulated to play a role in stationary phase PG stabilization whereas DacA is thought to aid regulation of crosslinking and maintenance of cell shape in exponential phase (22). Similar to *dacA* and *dacC*, two genes encoding amidases *amiC* and *amiD* which cleave the pentapeptide chain from the sugar backbone of PG were oppositely regulated in our dataset. AmiC in *E. coli* plays the largest role in PG degradation and remodelling during cell division and septation (26). The role of AmiD is poorly understood although our data suggest a novel role in stationary phase.

Taken together, stationary phase gene expression leads to more LD-crosslinks and more crosslinks to the OM via LPP, both contributing to decreasing envelope permeability.

***Outer membrane***

The phospholipid composition of the inner leaflet of the OM is known to change in stationary phase, with an increased cardiolipin concentration required for viability in *E. coli* (27)*.* Cardiolipin synthase B (*clsB*) was upregulated at 5 h in our dataset.

Genes involved in lipid A biosynthesis had decreased expression at 5 hours, suggesting that stationary phase lipid A production is downregulated, likely due to decreased growth. An increase in LPS in stationary phase has previously been linked to increased cell death (28).

Most genes involved in lipid A modification that were significantly altered in this dataset had decreased expression at 5 hours compared to 1 hour. These include genes responsible for resistance to cationic antimicrobial peptides including polymyxin (29). The addition of 4-amino-4-deoxy-L-arabinose (l-Ara4N; *arn* genes) and ethanolamine (*eptA*) to the phosphate groups of lipid A reduces its net negative charge (29). Similar negative charge-reducing modifications occur in the oligosaccharide core of LPS (30) and have an impact on CAMP resistance (31).

Palmitate incorporation into lipid A by PagP also confers resistance to CAMPs (although not polymyxin B) by increasing OM hydrophobicity. The PmrAB two-component regulatory system is the key regulator of LPS modification and polymyxin resistance (29) and is also downregulated at 5h in our dataset. It therefore appears that these LPS modifications are more prevalent in exponential growth and not required in stationary phase. Finally, a number of genes encoding O-antigen synthesis enzymes are downregulated at 5h; average O-antigen chain length is known to increase in stationary phase (32) and O-antigen structure has been shown to influence serum resistance (32) and CAMP susceptibility (33).

Only one lipid A modification gene was upregulated at 5 hours: *lpxO* encodes a dioxygenase that hydroxylates a myristoyl chain in lipid A (34). Deletion of *lpxO* in *S*. Typhimurium decreases survival in macrophages (35) and in *Klebsiella pneumonia*, an *lpxO* mutant is more sensitive to CAMPs (36).

Enterobacterial common antigen (ECA) when linked to LPS or in its cyclic form has been linked to envelope integrity and bile resistance (37–39). Six genes involved in the biosynthesis of ECA had decreased expression at 5h. It has previously been shown ECA is not converted to cyclic ECA in mutants lacking WecA, WecF or WecG (40), and as expression of the *wecG* decreased, it may be deduced that there is less cyclic ECA and less ECA in general in stationary phase.

***Regulatory networks***

Supplementary Table S1 also outlines the known regulation of genes differentially regulated in our RNASeq dataset, either in *Salmonella* or *E. coli*. Our EtBr accumulation data in Fig 5 suggested that RpoS only plays a minor role in envelope remodeling giving rise to decreased EtBr influx. In contrast, RpoS is seen to play a major role in envelope remodeling leading to increased SDS resistance in *E. coli* (41); however, this points to differences in regulation of envelope remodeling leading to different resistance phenotypes. The increase in CAMP resistance of stationary-phase *Salmonella* is RpoS-independent and instead partially dependent on PhoPQ (42). Indeed, PhoPQ has been shown to be a key regulator of envelope barrier function (43); influx of three dyes into stationary phase *S.* Typhimurium was greater in the absence than the presence of PhoP. The PmrAB regulon was seen to decrease in expression at 5 h. PmrA coordinates LPS modifications to reduce sensitivity to CAMPs (44), but our data suggests that these modifications are far more important in exponential phase, aligning with our ‘division of labour’ model in stationary phase whereby each layer of the envelope plays a larger role in barrier function.

1. Bergmiller T, Andersson AMC, Tomasek K, Balleza E, Kiviet DJ, Hauschild R, Tkačik G, Guet CC. 2017. Biased partitioning of the multidrug efflux pump AcrAB-TolC underlies long-lived phenotypic heterogeneity. Science (80- ) 356:311–315.

2. Datsenko KA, Wanner BL. 2000. One-step inactivation of chromosomal genes in *Escherichia coli* K-12 using PCR products. Proc Natl Acad Sci U S A 97:6640–5.

3. Cherepanov PP, Wackernagel W. 1995. Gene disruption in *Escherichia coli*: TcR and KmR cassettes with the option of Flp-catalyzed excision of the antibiotic-resistance determinant. Gene 158:9–14.

4. Bianco CM, Fröhlich KSA, Vanderpoola CK. 2019. Bacterial cyclopropane fatty acid synthase mRNA is targeted by activating and repressing small RNAs. J Bacteriol 201:e00461-19.

5. Qi Y, Liu H, Chen X, Liu L. 2019. Engineering microbial membranes to increase stress tolerance of industrial strains. Metab Eng 53:24–34.

6. Huisman GW, Siegele D a, Zambrano MM, Kolter R. 1996. Morphological and physiological changes during stationary phase. Escherichia coli Salmonella Cell Mol Biol 70:1545–1554.

7. Wang A ‐Y, Cronan JE. 1994. The growth phase‐dependent synthesis of cyclopropane fatty acids in *Escherichia coli* is the result of an RpoS(KatF)‐dependent promoter plus enzyme instability. Mol Microbiol 11:1009–17.

8. Kim BH, Kim S, Kim HG, Lee J, Lee IS, Park YK. 2005. The formation of cyclopropane fatty acids in *Salmonella enterica* serovar Typhimurium. Microbiology 151:209–18.

9. Chang YY, Cronan JE. 1999. Membrane cyclopropane fatty acid content is a major factor in acid resistance of *Escherichia coli*. Mol Microbiol 33:249–259.

10. Chen YY, Gänzle MG. 2016. Influence of cyclopropane fatty acids on heat, high pressure, acid and oxidative resistance in *Escherichia coli*. Int J Food Microbiol 222:16–22.

11. Mengin-Lecreulx D, Van Heijenoort J. 1985. Effect of growth conditions on peptidoglycan content and cytoplasmic steps of its biosynthesis in *Escherichia coli*. J Bacteriol 163:208–212.

12. Glauner B, Holtje J V., Schwarz U. 1988. The composition of the murein of *Escherichia coli*. J Biol Chem 263:10088–10095.

13. Morè N, Martorana AM, Biboy J, Otten C, Winkle M, Serrano CKG, Montón Silva A, Atkinson L, Yau H, Breukink E, den Blaauwen T, Vollmer W, Polissi A. 2019. Peptidoglycan remodeling enables *Escherichia coli* to survive severe outer membrane assembly defect. MBio 10:e02729-18.

14. Magnet S, Dubost L, Marie A, Arthur M, Gutmann L. 2008. Identification of the L,D-transpeptidases for peptidoglycan cross-linking in *Escherichia coli*. J Bacteriol 190:4782–4785.

15. Weber H, Polen T, Heuveling J, Wendisch VF, Hengge R. 2005. Genome-wide analysis of the general stress response network in *Escherichia coli*: σS-dependent genes, promoters, and sigma factor selectivity. J Bacteriol 187:1591–603.

16. Traxler MF, Summers SM, Nguyen HT, Zacharia VM, Hightower GA, Smith JT, Conway T. 2008. The global, ppGpp-mediated stringent response to amino acid starvation in *Escherichia coli*. Mol Microbiol 68:1128–1148.

17. Sanders AN, Pavelka MS. 2013. Phenotypic analysis of *Eschericia coli* mutants lacking L,D-transpeptidases. Microbiol (United Kingdom) 159:1842–1852.

18. Goffin C, Ghuysen J-M. 2002. Biochemistry and Comparative Genomics of SxxK Superfamily Acyltransferases Offer a Clue to the Mycobacterial Paradox: Presence of Penicillin-Susceptible Target Proteins versus Lack of Efficiency of Penicillin as Therapeutic Agent. Microbiol Mol Biol Rev 66:702–738.

19. Peters K, Pazos M, Edoo Z, Hugonnet JE, Martorana AM, Polissi A, VanNieuwenhze MS, Arthur M, Vollmer W. 2018. Copper inhibits peptidoglycan LD-transpeptidases suppressing β-lactam resistance due to bypass of penicillin-binding proteins. Proc Natl Acad Sci U S A 115:10786–10791.

20. Asmar AT, Collet JF. 2018. Lpp, the Braun lipoprotein, turns 50—major achievements and remaining issues. FEMS Microbiol Lett 365.

21. Magnet S, Bellais S, Dubost L, Fourgeaud M, Mainardi JL, Petit-Frère S, Marie A, Mengin-Lecreulx D, Arthur M, Gutmann L. 2007. Identification of the L,D-transpeptidases responsible for attachment of the Braun lipoprotein to *Escherichia coli* peptidoglycan. J Bacteriol 189:3927–3931.

22. van Heijenoort J. 2011. Peptidoglycan Hydrolases of *Escherichia coli*. Microbiol Mol Biol Rev 75:636–63.

23. Singh SK, Saisree L, Amrutha RN, Reddy M. 2012. Three redundant murein endopeptidases catalyse an essential cleavage step in peptidoglycan synthesis of *Escherichia coli* K12. Mol Microbiol 86:1036–1051.

24. Lommatzsch J, Templin MF, Kraft AR, Vollmer W, Höltje AJV. 1997. Outer membrane localization of murein hydrolases: MltA, a third lipoprotein lytic transglycosylase in *Escherichia coli*. J Bacteriol 179:5465–5470.

25. Dik DA, Marous DR, Fisher JF, Mobashery S. 2017. Lytic transglycosylases: concinnity in concision of the bacterial cell wall. Crit Rev Biochem Mol Biol.

26. Egan AJF, Errington J, Vollmer W. 2020. Regulation of peptidoglycan synthesis and remodelling. Nat Rev Microbiol 18:446–460.

27. Hiraoka S, Matsuzaki H, Shibuya I. 1993. Active increase in cardiolipin synthesis in the stationary growth phase and its physiological significance in *Escherichia coli*. FEBS Lett 336:970–983.

28. Sutterlin HA, Shi H, May KL, Miguel A, Khare S, Huang KC, Silhavy TJ. 2016. Disruption of lipid homeostasis in the Gram-negative cell envelope activates a novel cell death pathway. Proc Natl Acad Sci U S A 113:E1565–E1574.

29. Raetz CRH, Reynolds CM, Trent MS, Bishop RE. 2007. Lipid a modification systems in gram-negative bacteria. Annu Rev Biochem 76:295–329.

30. May JF, Groisman EA. 2013. Conflicting roles for a cell surface modification in *Salmonella*. Mol Microbiol 88:970–983.

31. Agrawal A, Weisshaar JC. 2018. Effects of alterations of the *E. coli* lipopolysaccharide layer on membrane permeabilization events induced by Cecropin A. Biochim Biophys Acta - Biomembr 1860:1470–1479.

32. Bravo D, Silva C, Carter JA, Hoare A, Álvarez SA, Blondel CJ, Zaldívar M, Valvano MA, Contreras I. 2008. Growth-phase regulation of lipopolysaccharide O-antigen chain length influences serum resistance in serovars of *Salmonella*. J Med Microbiol 57:938–46.

33. Ricci V, Zhang D, Teale C, Piddock LJV. 2020. The o-antigen epitope governs susceptibility to colistin in *Salmonella enterica*. MBio 11:2831–19.

34. Fernández PA, Velásquez F, Garcias-Papayani H, Amaya FA, Ortega J, Gómez S, Santiviago CA, Álvarez SA. 2018. Fnr and ArcA regulate lipid A hydroxylation in *Salmonella enteritidis* by controlling *lpxO* expression in response to oxygen availability. Front Microbiol 9:1220.

35. Moreira CG, Herrera CM, Needham BD, Parker CT, Libby SJ, Fang FC, Trent MS, Sperandio V. 2013. Virulence and stress-related periplasmic protein (VisP) in bacterial/host associations. Proc Natl Acad Sci U S A 110:1470–1475.

36. Llobet E, Martínez-Moliner V, Moranta D, Dahlström KM, Regueiro V, Tomása A, Cano V, Pérez-Gutiérrez C, Frank CG, Fernández-Carrasco H, Insua JL, Salminen TA, Garmendia J, Bengoechea JA. 2015. Deciphering tissue-induced *Klebsiella pneumoniae* lipid a structure. Proc Natl Acad Sci U S A 112:E6369–E6378.

37. Klobucar K, French S, Côté JP, Howes JR, Brown ED. 2020. Genetic and chemical-genetic interactions map biogenesis and permeability determinants of the outer membrane of *Escherichia coli*. MBio 11:e00161-20.

38. Mitchell AM, Srikumar T, Silhavy TJ. 2018. Cyclic enterobacterial common antigen maintains the outer membrane permeability barrier of *Escherichia coli* in a manner controlled by YhdP. MBio 9:e01321-18.

39. Rai AK, Mitchell AM. 2020. Enterobacterial common antigen: Synthesis and function of an enigmatic molecule. MBio.

40. Erbel PJA, Barr K, Gao N, Gerwig GJ, Rick PD, Gardner KH. 2003. Identification and biosynthesis of cyclic enterobacterial common antigen in *Escherichia coli*. J Bacteriol 185:1995–2004.

41. Mitchell AM, Wang W, Silhavy TJ. 2017. Novel RpoS-dependent mechanisms strengthen the envelope permeability barrier during stationary phase. J Bacteriol 199.

42. McLeod GI, Spector MP. 1996. Starvation- and stationary-phase-induced resistance to the antimicrobial peptide polymyxin B in *Salmonella typhimurium* is RpoS (σ(S)) independent and occurs through both *phoP*-dependent and -independent pathways. J Bacteriol 178:3683–8.

43. Murata T, Tseng W, Guina T, Miller SI, Nikaido H. 2007. PhoPQ-mediated regulation produces a more robust permeability barrier in the outer membrane of *Salmonella enterica* serovar typhimurium. J Bacteriol 189:7213–22.

44. Tamayo R, Prouty AM, Gunn JS. 2005. Identification and functional analysis of *Salmonella enterica* serovar Typhimurium PmrA-regulated genes. FEMS Immunol Med Microbiol 43:249–58.
